# *Staphylococcus aureus* Stress Response to Bicarbonate Depletion

**DOI:** 10.3390/ijms25179251

**Published:** 2024-08-26

**Authors:** Elisa Liberini, Sook-Ha Fan, Arnold S. Bayer, Christian Beck, Jacob Biboy, Patrice François, Joe Gray, Katharina Hipp, Iris Koch, Andreas Peschel, Brigitte Sailer, Daniela Vollmer, Waldemar Vollmer, Friedrich Götz

**Affiliations:** 1 Microbial Genetics, Interfaculty Institute of Microbiology and Infection Medicine Tübingen (IMIT), University of Tübingen, 72076 Tübingen, Germany; elisa@liberini.de (E.L.); fanfsh7@gmail.com (S.-H.F.); 2 The Lundquist Institute, Torrance, CA 90502, USA; 3 David Geffen School of Medicine at UCLA–University of California, Los Angeles, CA 90095, USA; 4 Department of Infection Biology, Interfaculty Institute of Microbiology and Infection Medicine Tübingen (IMIT), University of Tübingen, 72076 Tübingen, Germany; c.beck@uni-tuebingen.de (C.B.); andreas.peschel@uni-tuebingen.de (A.P.); 5 Biosciences Institute, Centre for Bacterial Cell Biology, Newcastle University, Newcastle upon Tyne NE1 7RU, UK; jacob.biboy@newcastle.ac.uk (J.B.); joe.gray@newcastle.ac.uk (J.G.); daniela.vollmer@newcastle.ac.uk (D.V.); waldemar.vollmer@newcastle.ac.uk (W.V.); 6 Genomic Research Laboratory, Division of Infectious Diseases, Faculty of Medicine, Geneva University Hospitals, 1205 Geneva, Switzerland; pf.patrice.francois@gmail.com; 7 Electron Microscopy Facility, Max-Planck-Institute for Biology, 72076 Tübingen, Germany; katharina.hipp@tuebingen.mpg.de (K.H.); iris.koch@tuebingen.mpg.de (I.K.); brigitte.sailer@tuebingen.mpg.de (B.S.); 8 Excellence Cluster 2124 ‘Controlling Microbes to Fight Infections’ (CMFI), University of Tübingen, 72076 Tübingen, Germany; 9 Institute for Molecular Bioscience, The University of Queensland, Brisbane, QLD 4072, Australia

**Keywords:** *Staphylococcus aureus*, bicarbonate transporter, MpsABC, cell wall, peptidoglycan, transcriptome

## Abstract

Bicarbonate and CO_2_ are essential substrates for carboxylation reactions in bacterial central metabolism. In *Staphylococcus aureus*, the bicarbonate transporter, MpsABC (membrane potential-generating system) is the only carbon concentrating system. An *mpsABC* deletion mutant can hardly grow in ambient air. In this study, we investigated the changes that occur in *S. aureus* when it suffers from CO_2_/bicarbonate deficiency. Electron microscopy revealed that Δ*mpsABC* has a twofold thicker cell wall thickness compared to the parent strain. The mutant was also substantially inert to cell lysis induced by lysostaphin and the non-ionic surfactant Triton X-100. Mass spectrometry analysis of muropeptides revealed the incorporation of alanine into the pentaglycine interpeptide bridge, which explains the mutant’s lysostaphin resistance. Flow cytometry analysis of wall teichoic acid (WTA) glycosylation patterns revealed a significantly lower α-glycosylated and higher ß-glycosylated WTA, explaining the mutant’s increased resistance towards Triton X-100. Comparative transcriptome analysis showed altered gene expression profiles. Autolysin-encoding genes such as *sceD*, a lytic transglycosylase encoding gene, were upregulated, like in vancomycin-intermediate *S. aureus* mutants (VISA). Genes related to cell wall-anchored proteins, secreted proteins, transporters, and toxins were downregulated. Overall, we demonstrate that bicarbonate deficiency is a stress response that causes changes in cell wall composition and global gene expression resulting in increased resilience to cell wall lytic enzymes and detergents.

## 1. Introduction

Bicarbonate and CO_2_ are two major forms of dissolved inorganic carbon (DIC) that serve as essential substrates for many metabolic processes in bacteria. Unlike autotrophic bacteria which can directly fix CO_2_, nonautotrophic bacteria rely on external sources of bicarbonate/CO_2_ for anaplerotic reactions. Bicarbonate plays a crucial role in the central metabolism of these bacteria because it is a substrate for biotin carboxylase which is part of the multi-enzyme complex, acetyl-CoA carboxylase, that catalyzes the first step in fatty acid synthesis [1,2]. Other carboxylases such as phosphoenolpyruvate carboxylase, pyruvate carboxylase, acetyl-CoA carboxylase, among others, play a vital role in central metabolism such as fueling the TCA cycle or biosynthesis of amino acids or nucleic bases like uracil and adenine [3,4]. Given the importance of CO_2_/bicarbonate, bacteria have evolved specialized mechanisms to concentrate these molecules to ensure a constant supply to cells. In numerous organisms, the enzyme carbonic anhydrase (CA) can rapidly convert CO_2_ to bicarbonate (HCO_3_^−^), thereby capturing it in the cytoplasm. In contrast to CO_2_, bicarbonate cannot freely diffuse in and out of the cell, and thus requires dedicated transporters to facilitate its movement across the cell membrane [5]. In cyanobacteria, three families of bicarbonate transporters have been identified: SbtA, a high-affinity sodium-dependent symporter; BicA, a low-affinity, high-flux sodium-dependent symporter; and BCT1, a high-affinity four-subunit transporter belonging to the ATP-binding cassette family [6,7]. Recently, a novel two-component transporter that facilitates DIC uptake was described in the chemolithoautotroph *Hydrogenovibrio crunogenus*, and surprisingly, these homologs are also found in nonautotrophic pathogen *Staphylococcus aureus* [8,9]. In *S. aureus*, the homologous bicarbonate transporter is encoded by the *mpsABC* operon, which was initially reported for its functionality as a membrane potential-generating system [10]. Radiolabeled NaH^14^CO_3_ uptake profiling showed that MpsABC represents a bicarbonate concentrating system, the first example of such a transporter in the phylum Bacillota [9]. As the only DIC supply system in *S. aureus*, MpsABC plays an important role in concentrating bicarbonate for key anaplerotic pathways, because CA is not present [11]. Deletion of *mpsABC* causes severe growth delay under ambient air conditions, which is reversible only by CO_2_ or bicarbonate supplementation. Of note, MpsABC is highly conserved and widespread in both autotrophic and nonautotrophic bacteria, suggesting its essential role in growth and survival [9,11,12]. BLASTp analysis of ~250 finished and ~4.500 unfinished *S. aureus* genomes revealed that MpsABC is the only DIC uptake mechanism in this species [11]. In addition, MpsABC has an advantage in species or strains where CO_2_ diffusion is hampered; for example, in mucus and biofilm-forming bacteria [12]. Recent studies of clinical Methicillin-Resistant *S. aureus* (MRSA) isolates revealed a novel phenotype, termed ‘NaHCO_3_ responsiveness’, in which a substantial proportion of MRSA strains exhibit enhanced susceptibility to β-lactams such as cefazolin and oxacillin in the presence of NaHCO_3_ [13]. It turned out that the NaHCO_3_ transporter MpsABC is a key contributor to the NaHCO_3_–β-lactam responsiveness phenotype in MRSA [14]. In this study, we aimed to investigate the significance of bicarbonate in Methicillin-Resistant *S. aureus* (MRSA) strain set JE2 in terms of phenotypic and transcriptomic changes by depleting the supply of bicarbonate to the cells. This was accomplished by deleting the *mpsABC* genes, which encode for the bicarbonate transporter, as it is the only source of bicarbonate entry into the cell. Here, we found by electron microscopy that the *mpsABC* deletion mutant (Δ*mpsABC*) has increased thickness in the cell wall (CW). Moreover, the mutant was largely inert to lysostaphin-induced cell lysis and non-ionic surfactant Triton X-100. Using liquid chromatography–tandem mass spectrometry (LC–MS/MS), we further analyzed the peptidoglycan (PG) fragments of the mutant digested with lysostaphin and cellosyl and detected the incorporation of alanine into the pentaglycine interpeptide bridge. We detected a lower content of α-glycosylated and higher content of ß-glycosylated wall teichoic acid (WTA) by flow cytometry. Comparative transcriptome analysis between the parent JE2 and mutant revealed altered gene expression profiles, particularly an upregulation of autolysins in the mutant.

## 2. Results

### 2.1. The mpsABC Deletion Mutant Has a Thicker Cell Wall

**Figure 1:**
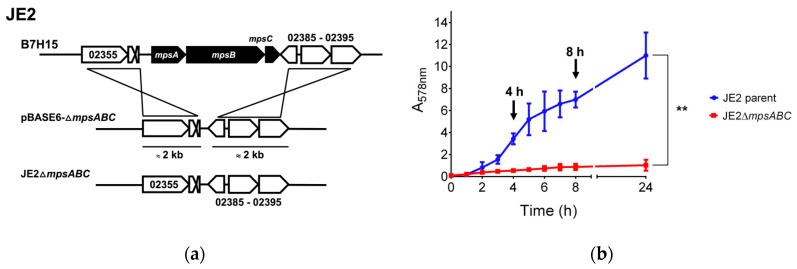
Deletion of *mpsABC* causes severe growth delay under ambient air. (**a**) Illustration of the construction of Δ*mpsABC* deletion mutant in the parent strain *S. aureus* JE2 (locus tag prefix: B7H15). For homologous recombination using allelic exchange, plasmid pBASE6-Δ*mpsABC* containing approximately 2 kb upstream and downstream DNA sequences of *mpsABC* was used. (**b**) Growth rates of Δ*mpsABC* were significantly lower than those of the parent JE2 strain (** *p* < 0.01 as determined by unpaired two-sided *t*-test). Arrows indicate the time points at 4 and 8 h when the samples were collected for transcriptomic analysis. Each time point in the graph represents the mean ± standard deviation (SD) from three independent biological replicates.

For our phenotypic studies, *mpsABC* was deleted in the background of a MRSA strain JE2, a well characterized and plasmid-free derivative of *S. aureus* USA300 LAC [15]. The deletion of *mpsABC* in JE2 (Δ*mpsABC*) (Figure 1a) resulted in an almost complete growth arrest under ambient air conditions (Figure 1b), as previously described for several other *S. aureus* strains [9,10]. After 24 h cultivation, the absorption at 578 nm (A_578_) of JE2Δ*mpsABC* was <1, whereas the JE2 parent strain reached its stationary phase at an A_578_ of >10. This growth defect could be rescued by aeration with 5% CO_2_ (Figure S1). Attempts to isolate a suppressor mutant of JE2Δ*mpsABC* that thrives under ambient air conditions have failed, consistent with our earlier reports that *S. aureus* lacks an alternative DICconcentrating mechanism [9,11,12].

**Figure 2:**
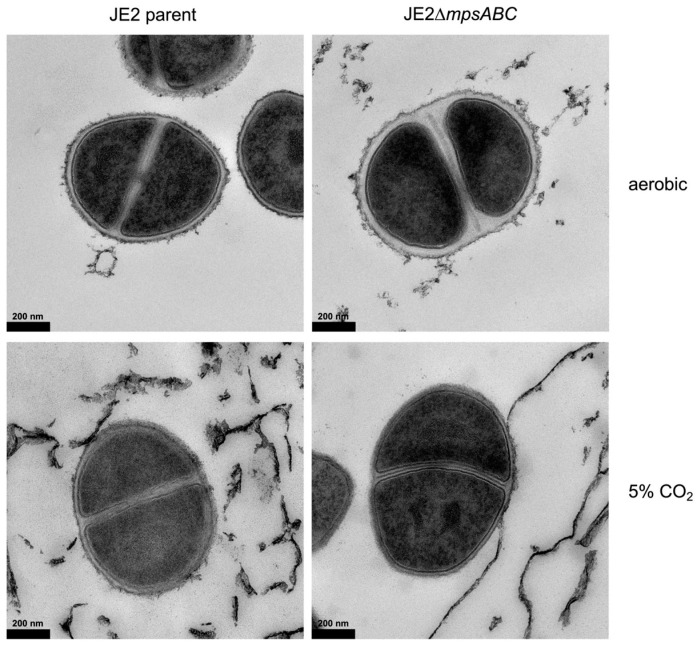
Cell wall (CW) of the JE2Δ*mpsABC* grown in ambient air is thicker than that of the parent strain. Transmission electron microscopy (TEM) images of JE2 parent and JE2Δ*mpsABC*. Cells were grown under ambient air and 5% CO_2_ at a starting A_578_ of 0.1, harvested at A_578_ 0.5 and washed with PBS pH 7.3 before fixation with 4% formaldehyde, 2.5% glutaraldehyde in 0.1 M phosphate buffer pH 7.4. The CW of JE2Δ*mpsABC* showed increased thickness. Scale bar is 200 nm.

Although the growth of the mutant was severely affected under ambient aeration conditions, it did not completely halt, which enabled us to perform transmission electron microscopy (TEM). The cells were cultivated under ambient air conditions until an A_578_ of 0.5, which took 2.5 h for the parental strain and 4.5 h for the mutant. TEM image analysis of more than 20 cells showed that the CW of the mutant grown in ambient air was approximately twice as thick compared to the parent strain (Figure 2 and Figure S2). In JE2, the average thickness was 15 nm, whereas in the mutant, it was 33 nm (Table 1). When the cells were cultured under 5% CO_2_, the CW diameter decreased from 15 to 12 nm in JE2 and from 33 to 15 nm in the mutant.

### 2.2. The ΔmpsABC Mutant Is Largely Inert to Cell Lysis Induced by Lysostaphin and Triton X-100

**Figure 3:**
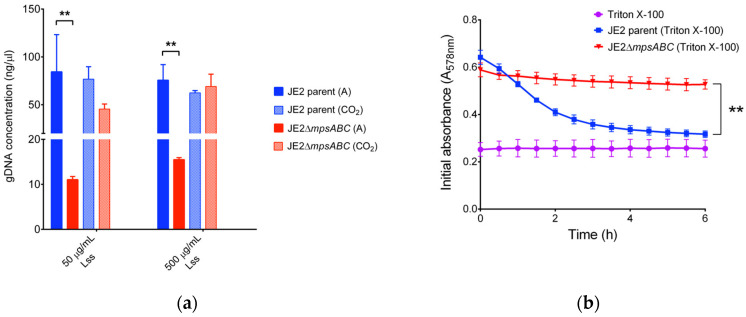
JE2Δ*mpsABC* cells are more resistant to lysis. (**a**) Concentration of genomic DNA (gDNA) released upon cell lysis from JE2 parent and its Δ*mpsABC* grown under ambient air and 5% CO_2_ following treatment with lysostaphin (Lss). Genomic DNA was isolated using the commercial Quick-DNATM Microprep Kit (ZYMO Research, Germany) according to the manufacturer’s protocol. Significantly less gDNA was isolated from the mutant than from the parent strain. Each bar in the chart shows the mean ± standard deviations (SD) from three independent biological replicates. Significance was calculated by two-way ANOVA with (** *p* < 0.01). The concentration of released gDNA upon cell lysis is listed in Table S1. (**b**) Triton X-100 induced autolysis assay. Triton X-100 (0.05% in PBS) was added at 0 h to mid-exponential phase cells. Autolysis was monitored by measuring the decrease in absorption (A_578_) every 30 min for 6 h (** *p* < 0.01 as determined by unpaired two-sided *t*-test). Triton X-100 alone served as negative control. Each value in the graph represents the mean ± standard deviations (SD) from three independent biological replicates.

When cells were exposed to CW-degrading lysostaphin, the mutant showed almost a 10-fold lower release of genomic (g) DNA upon cell lysis compared to the parental strain (Figure 3a and Table S1). The mutant also exhibited increased resistance to the non-ionic surfactant Triton X-100, while the same treatment in the parental strain led to a continuous decrease in absorption from A_578_ 0.65 to 0.3 over a 6 h period (Figure 3b).

### 2.3. The Peptidoglycan (PG) of the Mutant Contains Alanine in the Gly_5_-Bridge

**Figure 4:**
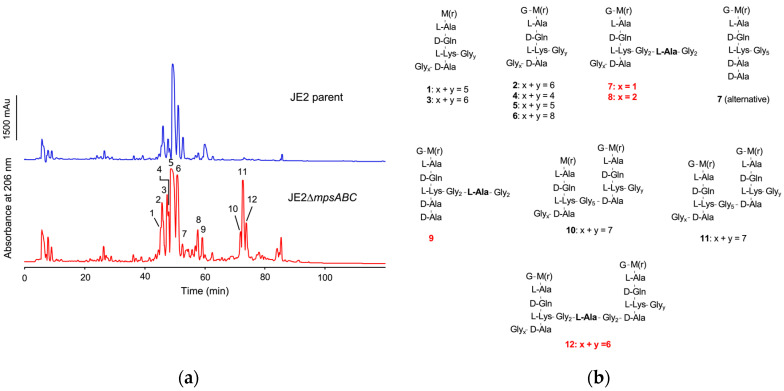
Muropeptides identified from lysostaphin-cellosyl PG digestion of JE2Δ*mpsABC* indicate the incorporation of L-alanine into the pentaglycine bridge. (**a**) Purified PG was digested with lysostaphin and cellosyl and the resulting profile of the JE2Δ*mpsABC* strain revealed distinct peaks at ~ 75–80 min retention time, which were absent in the PG digestion profile of the parent strain. (**b**) Collected peaks for lysostaphin-cellosyl-digested PG of JE2Δ*mpsABC* via LC-MS and LC-MS/MS analysis show incorporation of L-alanine into the pentaglycine bridge in the red highlighted peaks. The calculated masses are summarized in Table S3.

To determine the cause of the increased lysostaphin resistance in the mutant, we analyzed the composition of PG fragments by reversed-phase high-performance liquid chromatography (RP-HPLC) after PG digestion with cellosyl (muramidase from Streptomyces coelicolor), which yields crosslinked and non-crosslinked muropeptides [16]. PG digestion with cellosyl showed no significant differences in the composition between the JE2 parent and mutant (Figure S3). When PG was double digested with lysostaphin and cellosyl, peaks at a retention time of ~75 min were detected in the mutant profile only (Figure 4a). The muropeptide peaks of the mutant were analyzed by LC-MS and LC-MS/MS (Figure S4). The calculated masses of peak 7, 8, 9, and 12 were consistent with the incorporation of alanine into the pentaglycine interpeptide bridge (Gly_5_-bridge). Furthermore, peaks 10, 11, and 12 represented dimers that most likely could not be cleaved owing to the restricted activity of lysostaphin (Figure 4b, Table S3). In summary, PG double digestion with lysostaphin and cellosyl detected the incorporation of alanine into the Gly_5_-bridge (-Gly_x_-ala-Gly_y_-), explaining the increased lysostaphin resistance of the mutant. For determination of the PG crosslinking degree, we compared the area distribution of the mono- and oligomeric muropeptide peaks after mutanolysin digestion to the total peak area. The percentage distribution of muropeptides in the mutant did not change with respect to crosslinking compared to the parent strain (Table S2).

### 2.4. Comparative Transcriptome Analysis Revealed Differentially Regulated Genes in ΔmpsABC under Ambient Air Condition

To uncover possible genetic factors responsible for the growth defect observed in the mutant strain in ambient air and its thickened cell wall, we performed comparative transcriptome analysis using RNA-sequencing (RNA-seq). The mRNA of the parent and mutant strain was isolated after 4 and 8 h of growth (Figure 1b). While the parent strain was in the mid- or end-exponential phase, no clear growth phase could be determined for the mutant due to its very slow growth. The mutant preculture was cultivated in the presence of 5% CO_2_, which allowed initial growth due to remaining CO_2_/bicarbonate. However, the growth stopped after 2–3 h (Figure 1b). During this period, the mutant was presumably affected by CO_2_ limitation as revealed by gene expression analysis. The complete list of differentially expressed genes is summarized in Excel files: Table S5 (after 4 h), Table S6 (after 8 h), and Table S7 (5% CO_2_ condition). Table 2 comprises genes that were significantly up- or downregulated. Based on their function, we classified them into the following groups: CW lytic enzymes, wall teichoic acid (WTA) biosynthesis, CW-anchored proteins, secreted enzymes, transporters, toxins, regulators, and prophage genes. To validate the RNA-seq data, we performed qRT-PCR analysis of selected genes (Figure S5). RNA-seq and qRT-PCR data showed a high level of correlation. The comparison between the mutant strain and its parental strain after 4 h of growth revealed that many genes (838) were downregulated and only 87 genes were upregulated by at least a factor of 3 (Figure S6a). If we consider the cluster of orthologous groups (COG) of protein categories of the mutant, category U (intracellular trafficking and secretion) is 80%, category Q (secondary structure) is 70%, P (inorganic ion transport and metabolism) is 70%, E (amino acid metabolism and transport), and D (cell cycle control and mitosis) is about 70% and category H (coenzyme metabolism) is 100% downregulated. On the other hand, category O (posttranslational modification, protein turnover, chaperone function) with 80% is the highest expressed in the mutant. This might suggest that the mutant counteracts protein-folding deficits with upregulation of chaperons and foldases (Figure S6b).

### 2.5. The ΔmpsABC Mutant’s Wall Teichoic Acids (WTA) Showed a Lower α- and Higher ß-Glycosylation

**Figure 5:**
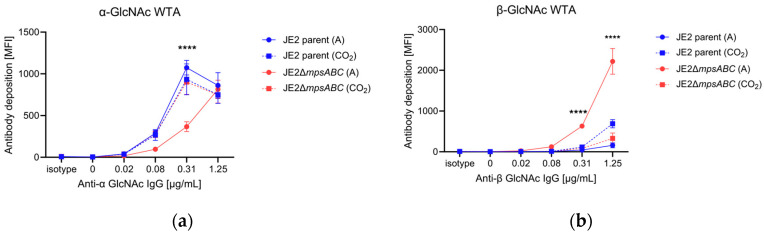
JE2Δ*mpsABC* has less α- and more ß-glycosylated GlcNAc WTA. Flow cytometry analysis of GlcNAc WTA (*N*-acetylglucosamine wall teichoic acid). Cells were grown in ambient air (A) or 5% CO_2_ (CO_2_) overnight, adjusted to A_578_ 0.4 and treated with 200 µg/mL proteinase K. Isotype control was anti-HIV protein gp120 (b12-IgG) Fab fragment (5 μg/mL). Secondary antibody was fluorescein isothiocyanate-labeled goat anti-human IgG F(ab′)2 FITC conjugate (2 μg/mL). (**a**) mAb (monoclonal antibody) 4461 Fab fragment against TarM dependent α-GlcNAc WTA. (**b**) mAb 4497 Fab fragment against TarS dependent ß-GlcNAc WTA. MFI: mean fluorescence intensity. Each value in the graph shows the mean ± standard error of the mean (SEM) from at least three independent biological replicates. Significance was calculated by two-way ANOVA with (**** *p* < 0.05).

In *S. aureus*, the WTA is glycosylated with GlcNAc either in the α- or β-configuration. TarM is the α-glycosyltransferase while TarS is a β-glycosyltransferase [17,18]. Transcriptome analysis revealed that *tarM* (α-1,4-glycosyltransferase) was approximately 40-fold downregulated in the Δ*mpsABC* mutant (Table 1). To see whether this is also associated with reduced α-glycosylation of the WTA, we analyzed the abundancy of α- and ß-glycosylated WTA by flow cytometry with Fab fragments targeting specific glycosylation patterns [19]. Indeed, we found less α-glycosylated WTA and significantly more ß-glycosylated WTA in the mutant strain compared to the parent strain or the mutant exposed to 5% CO_2_ during growth (Figure 5). This example shows that the genotype correlates well with the phenotype.

## 3. Discussion

In the present study, we observed significant phenotypic changes in the Δ*mpsABC* mutant following bicarbonate depletion. The most striking morphological difference between the mutant compared to the parent strain was the massively thickened CW (33 vs. 15 nm). Normally, the CW in *S. aureus* is approximately 20 nm thick [20,21]. However, CW thickness is not a fixed parameter but varies among strains, growth phase, medium, and the presence of antibiotics [20,21]. Inhibition of protein synthesis by antibiotics, such as chloramphenicol, puromycin, or actinomycin, can induce cell wall thickening beyond 100 nm [22,23]. The antibiotic-induced thickening of the CW is probably since CW synthesis continues independently of the inhibition of protein biosynthesis. We assume that bicarbonate depletion with its negative effects on the TCA cycle, respiration, and the synthesis of amino acids or other building blocks has a similar effect to the inhibition of translation by antibiotics. Berscheid et al. observed *sceD* upregulation, CW thickening, lysostaphin- and Triton X-100 resistance in the vancomycin-resistant laboratory mutant strain *S. aureus* VC40 [24], which was generated by serial passages of *S. aureus* RN4220Δ*mutS* in the presence of vancomycin [25]. Based on the RNA-seq of samples from two timepoints (4 and 8 h), alterations of gene expression profiles were observed after 4 h of bacterial growth in ambient air (Table 2). Genes encoding predicted CW hydrolases, such as SceD, were highly upregulated (600-fold) and to a lesser extent, IsaA. These hydrolases show sequence identity and similarity, with *E. coli* soluble lytic transglycosylase (LT) Slt in their PFAM06737 domain and C-terminal part [26]. Other upregulated autolysins were SsaA (a prophage-encoded amidase), LytM (a glycine-glycine endopeptidase), and LysM. LysM has a PG-binding domain that is involved in bacterial PG synthesis and remodeling [27,28]. The mutant’s CW thickness (Figure 2), therefore, seems strongly related to *sceD* upregulation, which is further supported by the repeal of the mutant’s increased CW thickness as well as the upregulation of *sceD* when grown in 5% CO_2_ (Table S7). Like the vancomycin-resistant *S. aureus* VC40 mutant, the Δ*mpsABC* mutant displayed increased resistance to lysostaphin and detergent Triton X-100-induced lysis. Both phenotypes suggested structural alterations in PG. An explanation could be that the mutant’s PG exhibits reduced crosslinking, thereby increasing its susceptibility to cleavage by cellosyl. During standard growth conditions in rich medium, *S. aureus* PG is highly crosslinked (80–90%) [29,30,31]. To investigate the extent of crosslinking, we analyzed the ratio of muropeptide abundance generated after mutanolysin digestion which indicates potential alterations in crosslinking. However, there were no significant differences in the distribution of mono- to hexameric muropeptides between the mutant and parent PG under ambient air or 5% CO_2_ (Table S2). The double digestion of mutant PG with lysostaphin and cellosyl revealed new dimeric muropeptides (peaks 10, 11, and 12) and alanine incorporation into the Gly_5_–bridge (peaks 7, 8, 9, and 12) (Figure 4, Table S3). The detection of dimeric PG fragments suggests that they are not (or poorly) digested by lysostaphin, consistent with earlier observations of staphylococci with L-amino acids in peptide bridges [32,33]. In staphylococci, the pentaglycine bridge is sequentially built by FemABX using glycyl-charged tRNA [34]. Presumably, the *mpsABC* mutant incorporates alanine into the interpeptide bridges in a similar mechanism to the recently reported FmhA-FmhC-mediated incorporation of L-serine in different *S. aureus* strains [35]. We explicitly looked for upregulation of genes in our RNAseq analysis encoding the Fem-like factors FmhA and FmhC, which incorporate Gly-Ser dipeptides into the pentaglycine bridges [35]; however, we observed no change in expression. The lysostaphin producer *Staphylococcus simulans* biovar *staphylolyticus* incorporates serine in the Gly_5_-bridge by the ‘lysostaphin immunity factor’ (Lif) to become resistant to lysostaphin [36,37]. FmhA and FmhC are homologs of Lif [38]. Therefore, we hypothesize that the incorporation of alanine into the interpeptide bridge causes lysostaphin resistance. The mechanisms of L-alanine incorporation are not clear. Since we could not observe a differential gene regulation of *fmhA* and *fmhC*, a possible explanation could be that the FmhA/C substrate alanyl-tRNA is more abundant than Ser- or Gly-tRNA. Another CW structural change was the altered glycosylation of WTA in the mutant. In *S. aureus*, approximately 60% of the CW is composed of WTA [39], a polymer covalently linked to MurNAc via phosphodiester bonds. WTA consists of chains of ribitol-phosphate units, esterified with D-alanine and glycosylated with *N*-acetylglucosamine (GlcNAc). This esterification process and the glycosylation pattern is crucial in regulating the activity of autolytic enzymes; it contributes to the resistance to cationic antimicrobial compounds and impacts the recognition by human immune receptors [19,39,40,41,42]. In the mutant *tarM*, which encodes the WTA α-1,4-glycosyltransferase [18], it was significantly (>40-fold) downregulated. Indeed, we found that the α-1,4-glycosylation of WTA was significantly decreased in the mutant (Figure 5a) while the TarS-mediated ß-1,4-glycosylation was increased compared to the parental strain (Figure 5b). In *S. aureus*, *tarS* is highly conserved and most likely constantly expressed [39], and it was therefore not surprising that we did not observe much up- or downregulation of *tarS* in the mutant. We therefore assume that a depletion of the α-1,4-glycosylation in WTA is compensated by an increased ß-1,4-glycosylation by TarS. The increase of ß-1,4-glycosylation in VC40 has been described to be correlated with higher vancomycin and detergent resistance [24,43]. We therefore assume that the mutant’s increased WTA ß-glycosylation is responsible for its enhanced resistance to Triton X-100-induced autolysis. Apparently, the mutant’s CW structural response to CO_2_/bicarbonate depletion stress is comparable to vancomycin-induced resistance CW adaptations in VC40. RNA-seq revealed the significant upregulation of ATP-dependent chaperone gene *clpB*. ClpB is a disaggregase and a key member of a multi-chaperone system that efficiently inhibits and reverses protein aggregation [44]. We hypothesize that in the *mpsABC* mutant, translation is affected due to substrate limitation, leading to incomplete and misfolded proteins and the expression of stress response factors such as ClpB. Consistent with this hypothesis, we identified a high number of other, differently regulated genes whose products are involved in protein turnover. In addition, we observed an induction of prophages since most phage genes were upregulated. Genes that encode secreted proteins, transporters, and toxins were found to be downregulated. The reduced expression of toxins correlated with the previously observed decrease in virulence of the Δ*mpsABC* mutant [9]. Many of these genes are activated by global regulators such as Agr and SarA, which were found to be downregulated in our study. This is consistent with the hypothesis that the mutant, when experiencing CO_2_/bicarbonate depletion, adjusts its metabolism to prioritize energy conservation and reduce the expression of genes that are not essential for survival. Therefore, we speculated that the lack of CO_2_/bicarbonate might be compensated by the overexpression of genes encoding enzymes involved in fatty acid biosynthesis and carboxylases. Contrary to our expectations, the mutant is unable to partially compensate the CO_2_/bicarbonate deficiency by overexpression of carboxylases. However, there was a downregulation of the *pckA*, PEP carboxylase, by 5-fold after 4 h and 100-fold after 8 h (Table 1). PckA was first described in *S. aureus* by Scovill et al., 1996, and they found that a mutant grew poorly in the absence of glucose [45]. PckA lies in the junction between glycolysis and the TCA cycle. Reduced expression of *pckA* has far-reaching consequences: the TCA cycle is not sufficiently replenished with oxaloacetate which leads to its halt, the respiratory chain comes to a standstill, and consequently, the membrane potential collapses. This aligns with our first phenotypic characterization of Δ*mpsABC* [10] in which we found decreased respiration and membrane potential.

## 4. Materials and Methods

### 4.1. Bacterial Strains and Growth Conditions

The strains used in this study were *S. aureus* JE2 [15] and its *mpsABC* deletion mutant (JE2ΔmpsABC) from our previous study [12]. Bacteria were grown with aeration at 130 rpm in Tryptic Soy Broth (TSB) with a flask to medium ratio of 1:10 in baffled flasks, unless stated otherwise.

### 4.2. Growth Studies

Overnight cultures were inoculated at a starting A_578_ of 0.1. Main cultures were grown at 37 °C with continuous shaking under ambient air or 5% CO_2_ conditions. Aliquots were collected at 0 h, and subsequently every hour for up to 8 h, as well as after 24 h for A_578_ determination.

### 4.3. RNA Isolation, Library Construction, Sequencing, and Analysis

To obtain RNA, precultures of *S. aureus* JE2 and JE2Δ*mpsABC* were cultivated overnight under 5% CO_2_ conditions. The main cultures of each strain were inoculated at a starting A_578_ of 0.1 and grown under ambient air conditions. Aliquots of A_578_ 2.0 were collected at 4 and 8 h. Qiagen RNAprotect (Qiagen, Hilden, Germany) was added to the aliquots and washed once with Tris-EDTA buffer (TE, 10 mM Tris-HCL, 1 mM EDTA pH 8) followed by a 30 min digestion with lysostaphin (Sigma-Aldrich, St. Louis, MO, USA), mutanolysin (Sigma-Aldrich, St. Louis, USA), and achromopeptidase (Sigma-Aldrich) at 37 °C. Total RNA was extracted using RNeasy Mini kit (Qiagen, Hilden, Germany) according to the manufacturer’s instructions, followed by an on-column DNase digestion (Qiagen, Germany). RNA quantification was performed using a Qubit fluorimeter (Thermo Fisher Scientific, Waltham, MA, USA), and RNA integrity assessed using a BioAnalyzer (Agilent Technologies, Santa Clara, CA, USA). The Stranded Total RNA Ribo-Zero Plus kit from Illumina was used for library preparation, with 500 ng of total RNA as input. Library molarity and quality were assessed using the Qubit and Tapestation (DNA High sensitivity chip, Agilent Technologies). Libraries were sequenced on a NovaSeq 6000 Illumina sequencer using oriented single-reads of 50 nt, yielding a minimum of 50 million mapped reads per sample. Raw reads were trimmed using Trimmomatic v.0.36 with the following options: SLIDINGWINDOW:10:30 MINLEN:25. The obtained clean-reads and the reference *Staphylococcus aureus* strain JE2 genome (GenBank accession: CP020619.1) were aligned using BWA v.0.7.17-r1188 with the BWA-MEM algorithm and the following options: -M–p. The samtools suite v.1.8 was used to obtain the final sorted bam files used to generate the reads count table. The reads count table was transformed into an RPKM table. Statistical analysis was performed using DSeq2 https://yanli.shinyapps.io/DEApp/ (accessed on 26 July 2024) with the Single-factor Experiment option. The lists of differentially regulated genes were exported and annotated as text files. Raw expression data for these samples were submitted to the European Nucleotide Archive (ENA) database under project accession number PRJEB65327.

### 4.4. qRT-PCR Validation

For qRT-PCR validation of selected genes, bacteria were grown and total RNA was isolated as described for RNA sequencing. Total RNA was treated with Turbo DNAse (Invitrogen, Waltham, MA, USA) and reverse-transcribed using random hexamers to generate a cDNA library with SuperScript IV (Invitrogen, Waltham, MA, USA). Following RNA-seq analyses, five genes were selected from the overall list of differentially expressed genes for qRT-PCR validation, representing the highest and lowest expressed genes from various categories including (i) transporters (*esxA*), (ii) CW-bound protein (*sasD*), (iii) virulence (*hla*), and (iv) CW hydrolase/modification (*lytM* and *sceD*). The primers used to amplify the selected genes to determine their relative expression are listed in Table S4. qRT-PCR was performed using StepOne thermocycler (Thermo Fisher, Waltham, MA, USA) and analyzed with StepOne Software, version 2.3. The gene *gyrB* was used as a housekeeping gene to normalize transcript quantification. Relative gene expression was calculated using the 2^−ΔΔCT^ method from two independent biological replicates for each strain/condition and was performed in triplicate on at least two separate runs. Relative gene expression for the selected genes from strains grown under 5% CO_2_ conditions was normalized to its corresponding gene grown under ambient air, with the latter set equal to 1.0. 

### 4.5. Triton X-100-Induced Autolysis Assay

The autolysis assay was performed as previously described [46]. Overnight cultures of *S. aureus* JE2 and JE2Δ*mpsABC* were adjusted to A_578_ of 0.05 and 0.1, respectively, and grown in BM (1% soy peptone, 0.5% yeast extract, 0.5% NaCl, 0.1% glucose, and 0.1% K_2_HPO_4_, pH 7.2) until mid-exponential phase to A_578_ 0.5. Cells were centrifuged to remove the growth medium and then washed twice with phosphate-buffered saline (PBS) (pH 7.2), followed by a final wash with ice-cold sterile MilliQ water. The cells were resuspended in PBS or PBS containing 0.05% Triton X-100, respectively, to an A_578_ of 1. Each cell suspension (500 μL) was added to a 48-well microplate (Greiner Bio-One, Frickenhausen, Germany) and incubated at 37 °C with continuous shaking on a multiplate reader (Varioskan Lux, Thermo Scientific, Waltham, MA, USA). Autolysis was monitored as a decrease in A_578_ after every 30 min for 6 h. PBS and PBS containing 0.05% Triton X-100 served as negative controls.

### 4.6. Lysostaphin-Induced Lysis Assay

To evaluate the resistance of *S. aureus* JE2 parent and JE2Δ*mpsABC* towards the CW hydrolyzing enzyme lysostaphin, overnight cultures were adjusted to an A_578_ of 1. The cell suspensions were centrifuged for 5 min at 5000× *g* to collect the cell pellets. The pellet was resuspended in 100 µL TSB containing lysostaphin at concentrations of 50, and 500 µg/mL and incubated at 37 °C for 30 min. Subsequently, the gDNA was purified using Quick-DNA^TM^ Microprep Kit (Zymo Research, Freiburg, Germany). The gDNA concentration was measured using Nanodrop (NanoPhotometer^®^ NP80, Implen GmbH, Munich, Germany).

### 4.7. Transmission Electron Microscopy

Overnight cultures of *S. aureus* JE2 and JE2Δ*mpsABC* (grown in CO_2_) were inoculated in TSB at a starting A_578_ of 0.05 and 0.1, respectively. All cultures were grown under their respective conditions with shaking at 37 °C until they reached an A_578_ of 0.5. An amount of 1 mL aliquots of each culture were collected and pelleted by centrifugation at 1000× *g* for 10 min before being washed twice with 1 mL of PBS (137 mM NaCl, 2.7 mM KCl, 10 mM Na_2_HPO_4_, 1.8 mM KH_2_PO_4_, pH 7.3). The cells were pre-fixed with fixing solution (4% formaldehyde, 2.5% glutaraldehyde in 0.1 M PO_4_ at pH 7.4) for 90 min at room temperature. Cells were high-pressure frozen (HPF Compact 03, Engineering Office M. Wohlwend GmbH) in capillaries and freeze substituted (AFS2, Leica Microsystems GmbH, Wetzlar, Germany) with 2% OsO_4_ and 0.4% uranyl acetate in acetone as a substitution medium and embedded in Epon. Ultrathin sections were stained with uranyl acetate and lead citrate and analyzed with a Tecnai Spirit transmission electron microscope (Thermo Fisher Scientific, Waltham, MA, USA) operated at 120 kV.

### 4.8. Muropeptide Analysis

PG was prepared as previously described [47,48] and incubated with 15 µg cellosyl in 20 mM sodium phosphate pH 4.8 for 16 h at 37 °C with stirring. An additional 15 µg of cellosyl was added and the incubation was continued overnight. Enzymatic digestion was stopped by boiling the samples for 5 min at 100 °C in a dry heat block and soluble material was collected after centrifugation at 13,000× *g* for 10 min. The supernatant was reduced with sodium borohydride and the resulting muropeptides were separated by RP-HPLC using the conditions and gradients described previously [48]. PG was prepared as described previously [47,48] and incubated with lysostaphin (300 µg) in 20 mM NaPi, pH 6.0, for 24 h at 37 °C with stirring. Subsequently, cellosyl (Höchst AG, Frankfurt, Germany) (15 µg) was added and the sample was incubated for 16 h at 37 °C. An additional 15 µg of cellosyl was added and incubation was continued for 16 h. Enzymatic digestion was stopped by boiling the samples for 5 min at 100 °C in a dry heat block and the soluble material was collected after centrifugation at 13,000× *g* for 10 min. The supernatant was reduced with sodium borohydride and the resulting monomeric muropeptides were separated by RP-HPLC using a Prontosil (Bischoff, Leonberg, Germany) column (3 µm, particle size, 250 × 4.6 mm, 120 Å pore size) maintained at 52 °C. A 140 min linear gradient (0–100%) of 10 mM sodium phosphate pH 6.0 with 0.00065% NaN_3_ to 10 mM sodium phosphate pH 6.0 and 30% MeOH at a flow rate of 0.5 mL/min was used for separation of muropeptides [49].

### 4.9. Mass Spectrometry Analysis of Monomeric Muropeptides

For LC-MS/MS analysis [50], the collected fractions were reconstituted in 20 µL of 0.2% formic acid (aq.). Typically, 10 µL was injected onto a microbore RP-HPLC column (ACE 3 C18, 1.0 × 150 mm) flowing at 50 µL min-1 in a 1100 HPLC system (Agilent, Cheadle, UK) at 35 °C, with the first 7 min of eluate diverted to waste due to high salt content. Buffer A was composed of water containing 0.1% (*v*/*v*) formic acid and buffer B was acetonitrile containing 0.1% (*v*/*v*) formic acid. The following elution gradient was used: starting at 0% buffer B, increasing to 4% B at 10 min, then on to 5% B at 30 min, rising to 10% B at 53 min, the gradient was ramped to 50% B at 58 min, then on to 85% B at 63 min, followed by a 2 min hold at 85% B, and finally 15 min re-equilibration at 0% B. The total run time was 80 min. The HPLC column eluate was directed to the mass spectrometer (LTQ Ion Trap MS, Thermo) via an IonMaxTM electrospray ion source (Thermo Fisher Scientifc, Waltham, MA, USA). The settings for the ion source were spray voltage and capillary temperature values of 4200 V and 200 °C, respectively, together with a sheath gas flow of 16 (arb) and a sweep gas flow of 1.0 (arb). MS data were acquired in positive ion mode over the range of 200–2000 *m*/*z* in Triple Scan mode. The precursor scan (Enhanced scan rate) was immediately followed by an UltraZoom scan (lower = 3 *m*/*z* units, upper = 5 *m*/*z* units), and finally, MS/MS acquisition was performed using a normal scan rate, with activation Q = 0.25 and activation time = 30 ms (with wide band activation turned on). The minimum signal threshold was set at 500 counts, MS/MS isolation width set at 2 *m*/*z*, preferred charge state range was set at +1 to +3, and undetermined charge states were excluded. The resulting mass spectral data were analyzed using QualBrowserTM software, version 4.4.16.14 (Thermo Fisher Scientifc, Waltham, MA, USA).

### 4.10. Flow Cytometry Analysis with WTA-Detecting Antibodies

*S. aureus* JE2 and its *mpsABC* deletion mutant were grown overnight at 37 °C under ambient air and 5% CO_2_ condition. Cells were adjusted to an A_578_ of 0.4 in Tris-HCL buffer (pH 8) and treated with 200 μg/mL proteinase K for 1 h at 37 °C. Incubation was continued overnight at 4 °C and stopped on the next day by inactivation at 95 °C for 10 min followed by centrifugation at 5000× *g* and buffer exchange with PBS containing 0.1% bovine serum albumin. An amount of 25 μL of the diluted bacteria were incubated with 25 μL serial dilutions of mAb (monoclonal antibody) 4461 or mAb 4497 Fab fragments in a 96-well plate for 30 min at 4 °C [19,51,52]. The samples were subsequently washed, centrifuged, and incubated with fluorescein isothiocyanate-labeled goat anti-human IgG F(ab′)2 FITC conjugate (2 μg/mL) (Merck, AQ112F) for 20 min at 4 °C in the dark. Labeled bacteria were washed, centrifuged, and fixed with 1% paraformaldehyde for 20 min at room temperature in the dark. The bacteria were centrifuged again and resuspended in PBS, and surface-bound IgG Fab was measured by flow cytometry using a BD FACSCalibur. Anti-HIV protein gp120 (b12-IgG) Fab fragment (5 μg/mL) was used as isotype control. The whole bacterial population was gated, and the mean FL-1 fluorescence was analyzed with FlowJo version 10.8.1. The WTA-specific mAb 4461, mAb 4497, and the B12 isotype control were kindly provided by Prof. N. van Sorge (Amsterdam UMC, Amsterdam, The Netherlands) and described previously [19].

### 4.11. Statistical Analyses and Quantification of CW Thickness

All data are presented as sample mean ± SD, unless specified otherwise. All statistical analyses were performed using Student’s *t*-test or two-way ANOVA for comparison between groups using GraphPad Prism 9 software. *p*-values of < 0.05 were considered statistically ‘significant’. CW thickness was quantified using ImageJ-win64 [52] from a total of 20 cells for each strain under their respective growth conditions.

## 5. Conclusions

*S. aureus* serves as an exemplary model for mesophilic bacteria to study the stress response to bicarbonate depletion, as it relies solely on the MpsABC-type bicarbonate transporter for CO_2_ concentration. Adequate bicarbonate supply is crucial, evidenced by the inability of an *mpsABC* mutant to grow in ambient air. This mutant exhibits characteristics of viable but non-culturable (VBNC) cells, making it a valuable model for investigating bicarbonate-depletion stress responses. Observations of the *mpsABC* mutant revealed differential gene expression and cell wall (CW) modifications, including increased thickness, enhancing resilience to lytic enzymes and detergents. A thickened CW has previously been reported in *S. aureus* treated with translation-inhibiting antibiotics and more recently in a vancomycin-resistant mutant. This raises the question: what parallels between antibiotic stress and bicarbonate deficiency lead to similar stress responses? Both conditions impair protein synthesis and growth. However, CW synthesis can proceed independently of protein synthesis, allowing CW thickening despite growth arrest. This might explain the similar stress response to antibiotic stress and bicarbonate depletion.

## Figures and Tables

**Table 1:** CW thickness measurements of *S. aureus* JE2 parent and JE2Δ*mpsABC* exposed to ambient air and 5% CO_2_.

Cell Wall Thickness in nm (mean ± SD)		
Strain	Ambient Air	5% CO_2_
JE2 parent	15.37 ± 2.64	12.05 ± 2.44
JE2Δ*mpsABC*	33.43 ± 7.71	15.00 ± 2.78

Quantification of the CW thickness (nm) was determined using the program ImageJ (Fiji software, version 1.53c; ImageJ-win64). Each value represents the mean ± standard deviations (SD) from a total of 20 cells for each strain (JE2 parent and Δ*mpsABC*) in each condition (ambient air and 5% CO_2_).

**Table 2:** Selected up-/downregulated genes in Δ*mpsABC* vs. JE2 parent after 4 and 8 h.

Protein Number	Function	Fold Change Mutant vs. Parent	
		4 h	8 h
**Cell wall (CW) lytic enzymes**			
B7H15_11645	SceD, lytic transglycosylase	592.3	517.1
B7H15_14290	IsaA, lytic transglycosylase	4.3	2.1
B7H15_12775	SsaA, secretory antigen	20	5.2
B7H15_10875	Amidase, 251 aa, prophage-encoded	16.8	9.9
B7H15_01500	LytM, glycine-glycine endopeptidase	14.8	8.4
B7H15_03720	LysM, peptidoglycan-binding domain-containing protein, probably autolysin	8	2
**Wall teichoic acid (WTA) biosynthesis**			
B7H15_05355	TarM, poly(ribitol-phosphate) α-*N*-acetylglucosaminyltransferase	−43.3	−61.5
**CW anchored proteins**			
B7H15_00750	SasD, cell wall-anchored protein	−27.3	−3.2
B7H15_14775	SasA, serine-rich repeat glycoprotein adhesin	−23.4	−15
B7H15_13880	SIRK signal domain/LPXTG anchor domain surface protein	−22.2	−3.3
B7H15_00135	AdsA, LPXTG-anchored adenosine synthase	−11.5	−4.1
B7H15_00620	Spa, staphylococcal protein A	−6.4	−1.6
**Secreted enzymes**			
B7H15_01535	Peptidase C51 domain-containing protein	−64.6	−31
B7H15_14690	Aureolysin, zinc metalloproteinase	−44.6	−1.2
B7H15_00545	Phospholipase C, phosphatidylinositol	−23.1	−1.7
B7H15_01765	Lip2(geh), triacylglycerol lipase	−20.3	−200
**Transporters**			
B7H15_01540	Type VII secretion effector EsxA (and all other type VII secretion protein genes)	−56.5	−102
B7H15_01675	Formate/nitrite transporter family protein	−33.7	−1.7
B7H15_01120	Peptide ABC transporter substrate-binding protein	−15.3	−4.7
B7H15_01110	ABC transporter permease	−14.9	−6
B7H15_00420	YdhK family protein, lipoprotein, putative	−13.5	−29
B7H15_00415	Copper-translocating P-type ATPase	−11.2	−21.6
B7H15_03550	Metal ABC transporter permease	−11.2	−176
**Toxins**			
B7H15_02365	PSM-α3, phenol-soluble modulin	−479	−6360
B7H15_11280	δ-lysin, phenol-soluble modulin	−336	−11,215
B7H15_06120	PSM-ß, phenol-soluble modulin	−235	−5200
B7H15_06050	Hla, α-hemolysin	−61	−2408
**Regulators**			
B7H15_14830	IcaR, ica operon transcriptional repressor	−20.7	−15.6
B7H15_11300	AgrA, accessory gene regulator protein A	−25	−91
B7H15_11285	AgrB, accessory gene regulator protein B	−24	−89
B7H15_11290	AgrD, accessory gene regulator protein D	−24	−69
B7H15_11295	AgrC, accessory gene regulator protein C	−28	−90
B7H15_03430	SarA, staphylococcal accessory regulator	−1.7	−3
B7H15_06535	CodY, transcriptional repressor	−1.2	−1.6
**Prophage genes**			
B7H15_10915	Phage tail family protein (and most phage-related genes)	12.5	nd
B7H15_11045	PVL, phi PVL orf 51-like protein	16.5	13
**Cross bridge synthesis**			
B7H15_13360	FmhA, fem-like factor	1.02	1.7
B7H15_06500	FmhC, fem-like factor	0.98	−2.3
**Fatty acid biosynthesis**			
B7H15_06405	FabD, ACP S-malonyltransferase	1.2	1.5
B7H15_05075	FabF, ß-ketoacyl-ACP synthase II	−1.5	−1.2
B7H15_06410	FabG, 3-oxoacyl-acyl-carrier-protein reductase	−1.2	−1.1
B7H15_05210	FabI, enoyl-ACP reductase	1.5	1.7
B7H15_11660	FabZ, 3-hydroxyacyl-ACP dehydratase	2.2	1.6
B7H15_07130	PlsY, glycerol−3-phosphate acyltransferase	−1.2	−1.5
B7H15_06400	PlsX, phosphate acyltransferase	−1.5	1
B7H15_06385	FakA, fatty acid kinase	1.1	1
B7H15_04195	FakB1, fatty acid binding protein 1	−1.2	1
B7H15_07520	FakB2, fatty acid binding protein 2	1	1
**Carboxylating Enzymes**			
B7H15_09750	PckA, PEP carboxylase	−5	−100
B7H15_08395	AccC, acetyl-coA carboxylase	1.2	2

(−) indicates a downregulation of gene expression in the mutant compared to the parent. nd, not determined.

## Data Availability

Raw expression data for these samples were submitted to the ENA database under project accession number PRJEB65327.

## Supplementary Materials

The following supporting information can be downloaded at: https://www.mdpi.com/article/10.3390/ijms25179251/s1.
