# Supplementary material for: Staphylococcus aureus Stress Response to Bicarbonate Depletion
Source: Int J Mol Sci. 2024 Aug 26;25(17):9251. doi: 10.3390/ijms25179251 (PMC11394868; doi:10.3390/ijms25179251)
Supplement: Supplementary file 1 [file ijms-25-09251-s001.zip › Fig. S4.pdf]

A LC-MS Peak 1

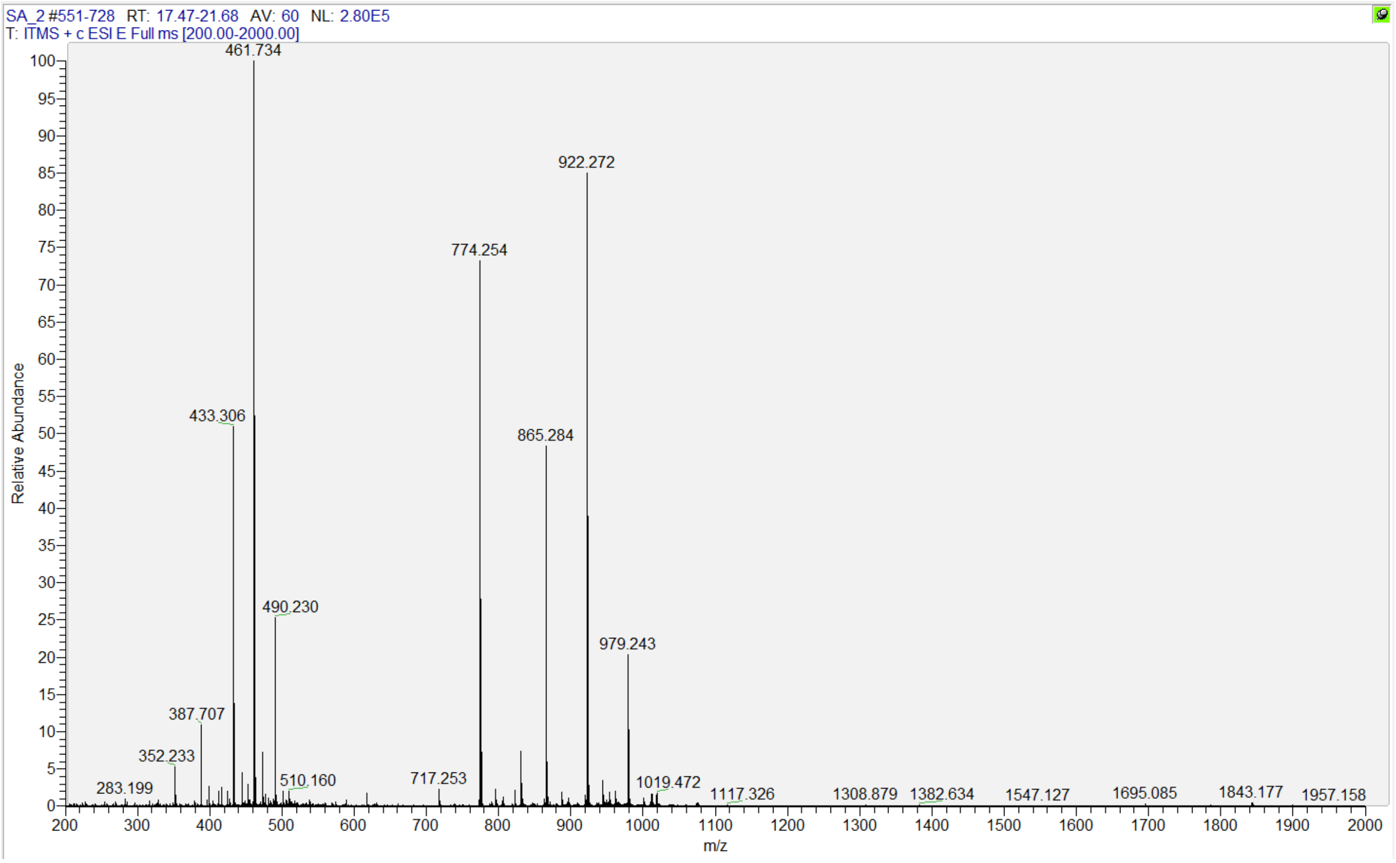

B LC-MS/MS Peak 1

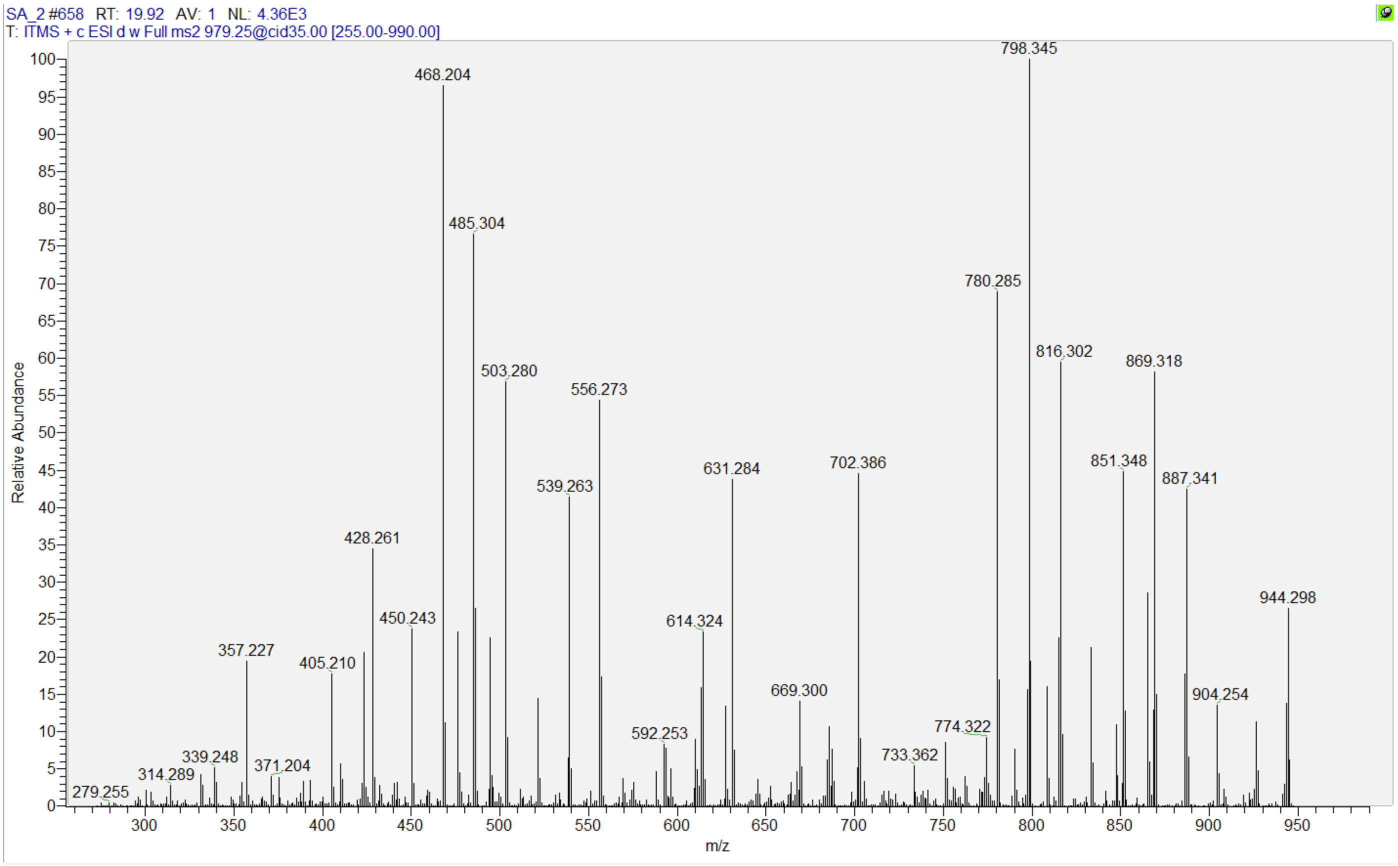

A LC-MS Peak 2

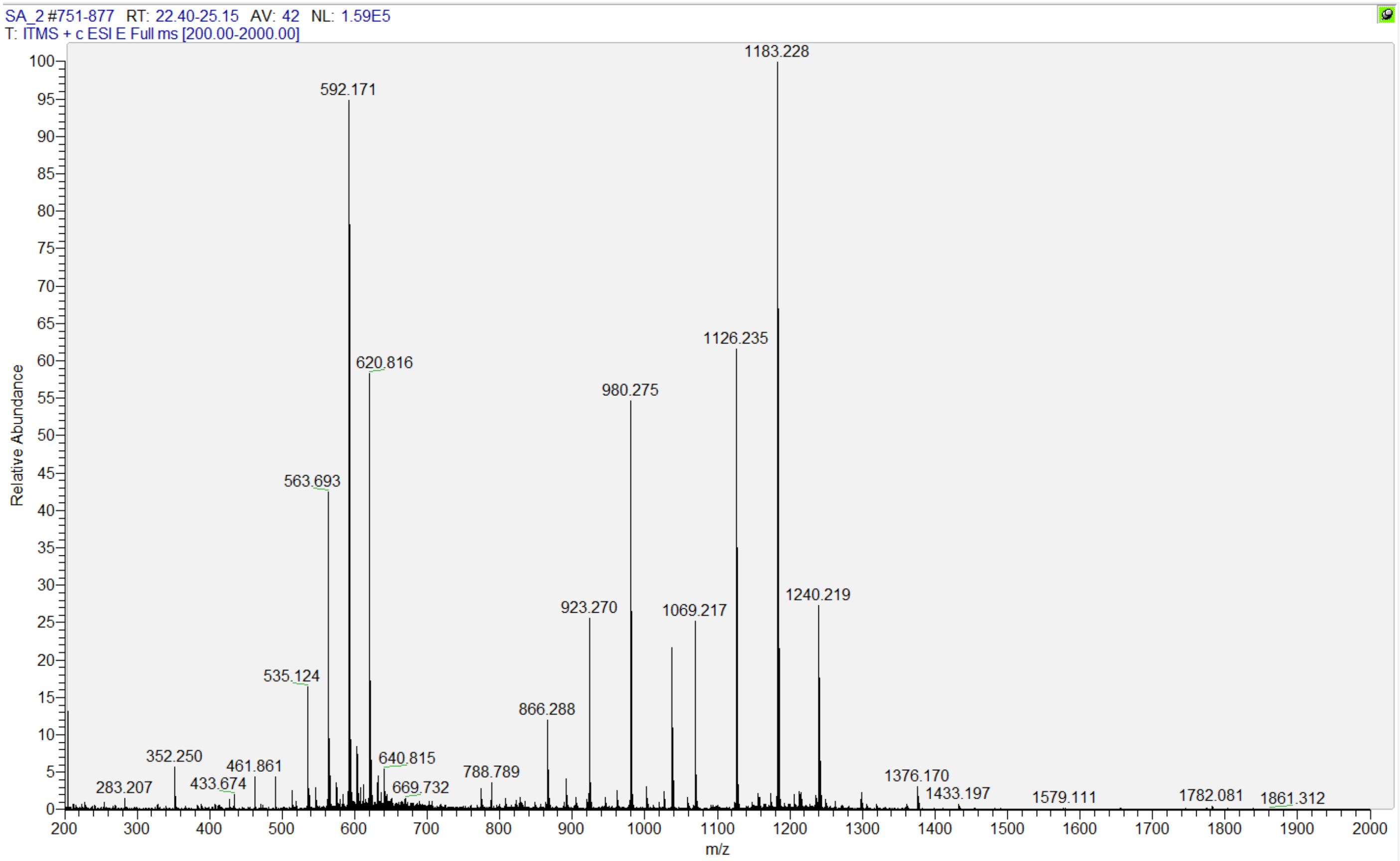

B LC-MS/MS Peak 2

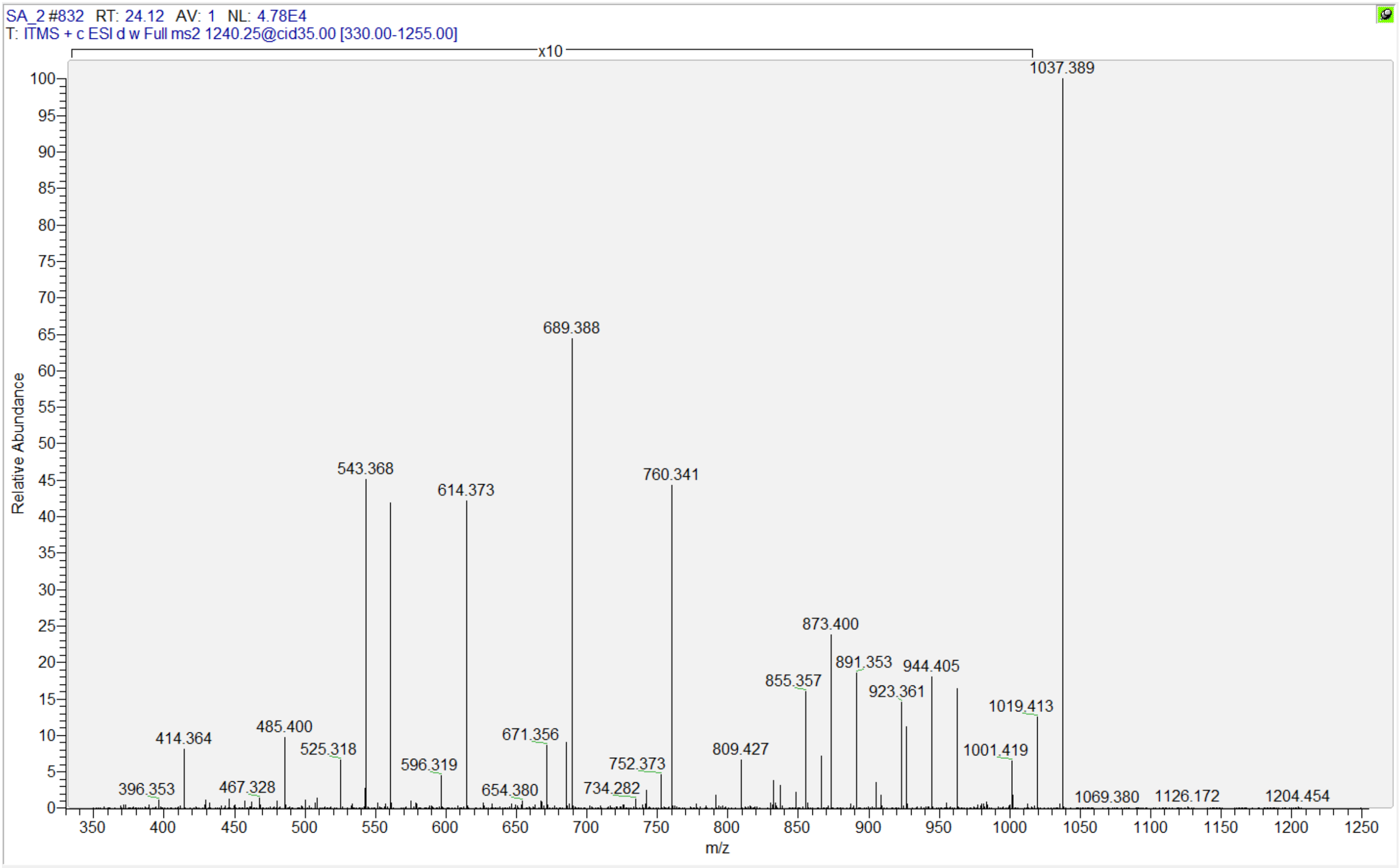

A LC-MS Peak 3

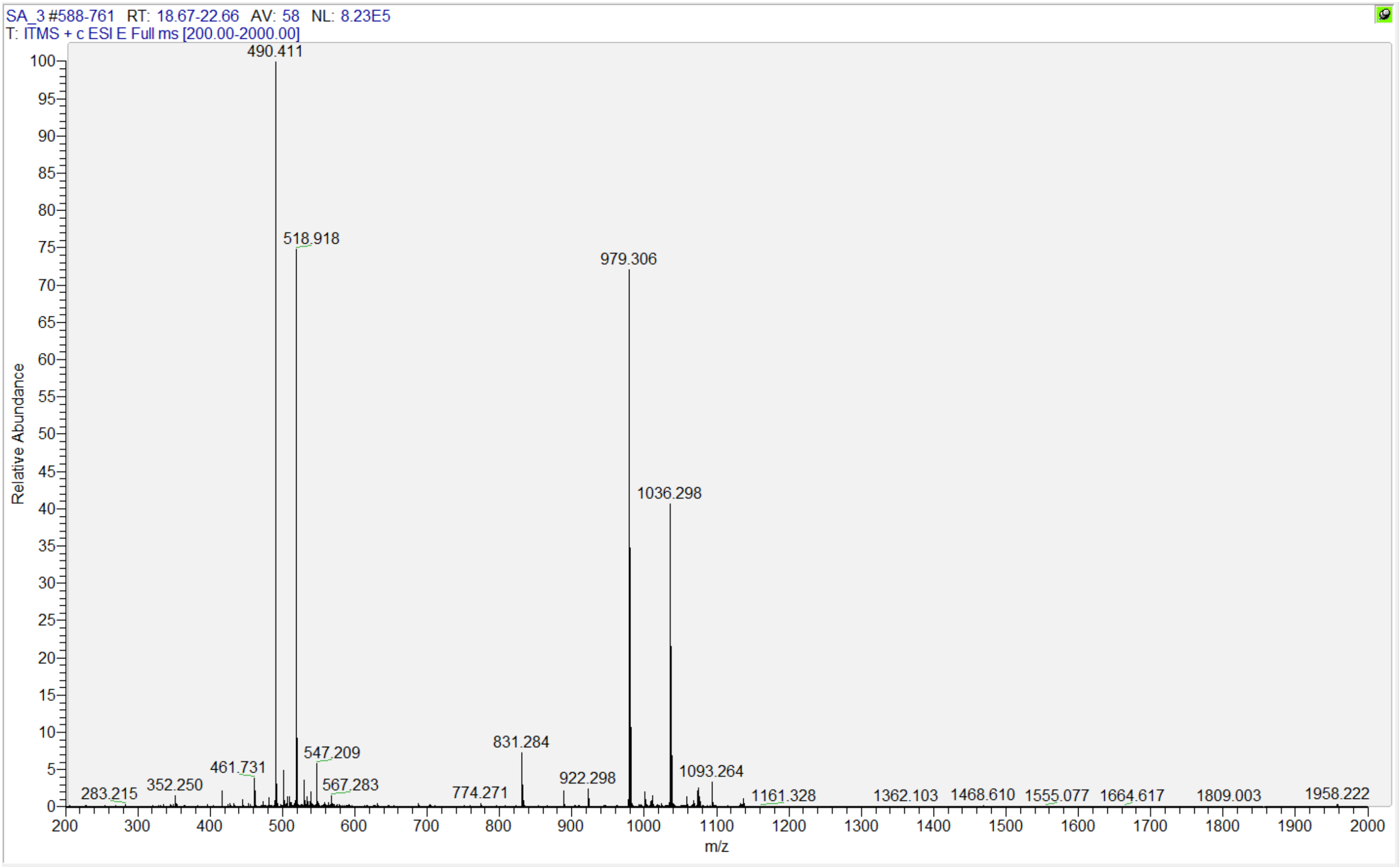

B LC-MS/MS Peak 3

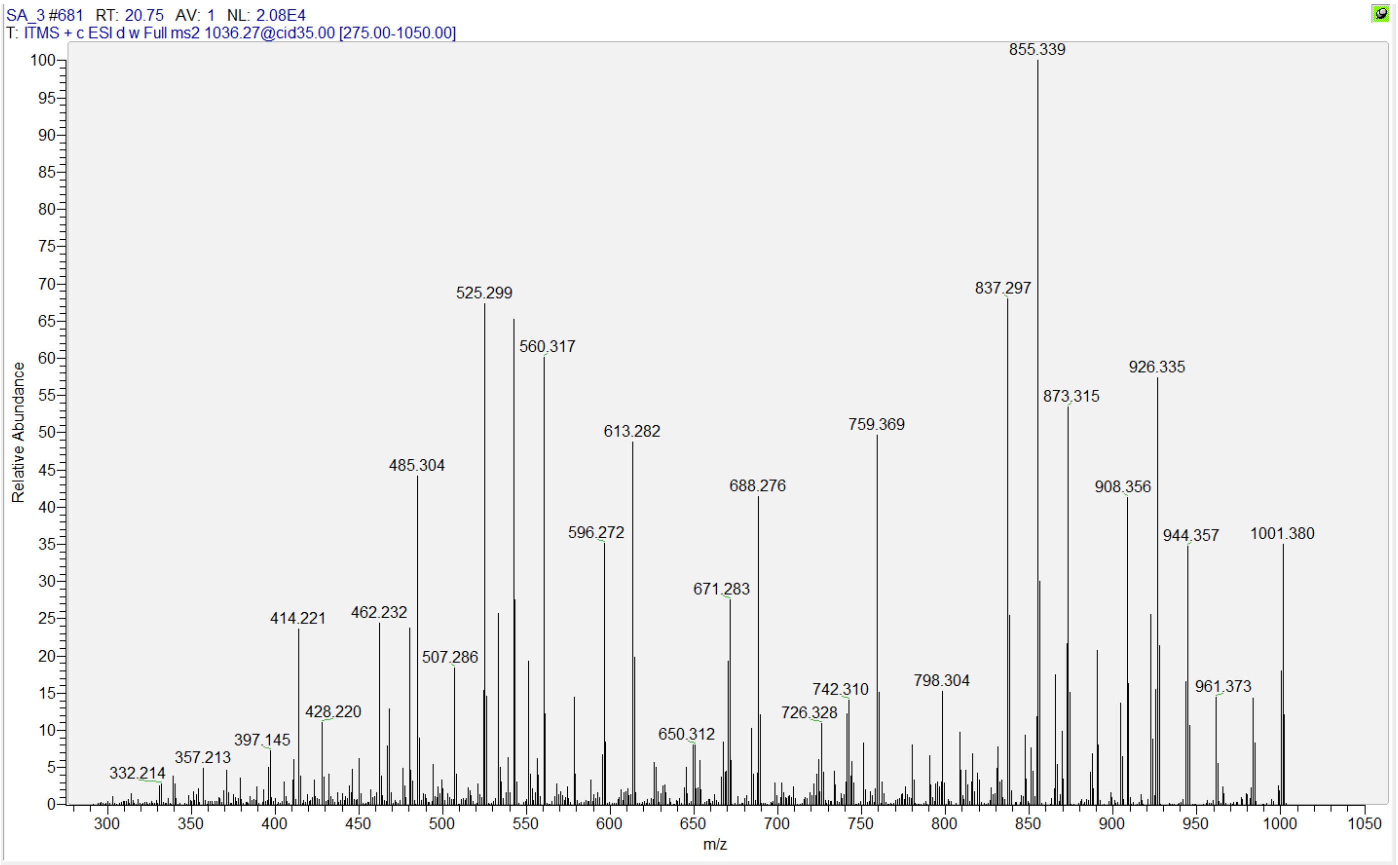

A LC-MS Peak 4

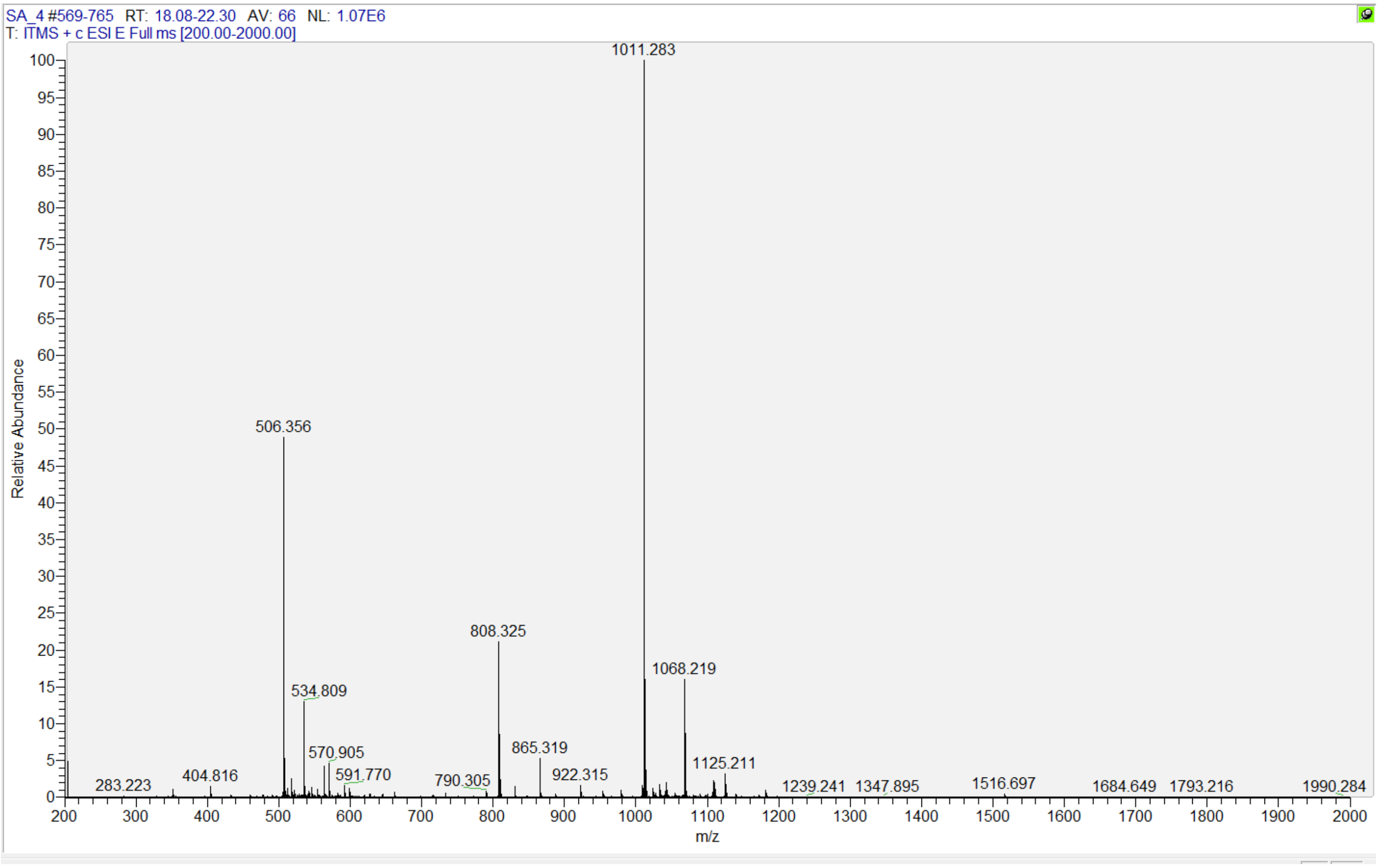

B LC-MS/MS Peak 4

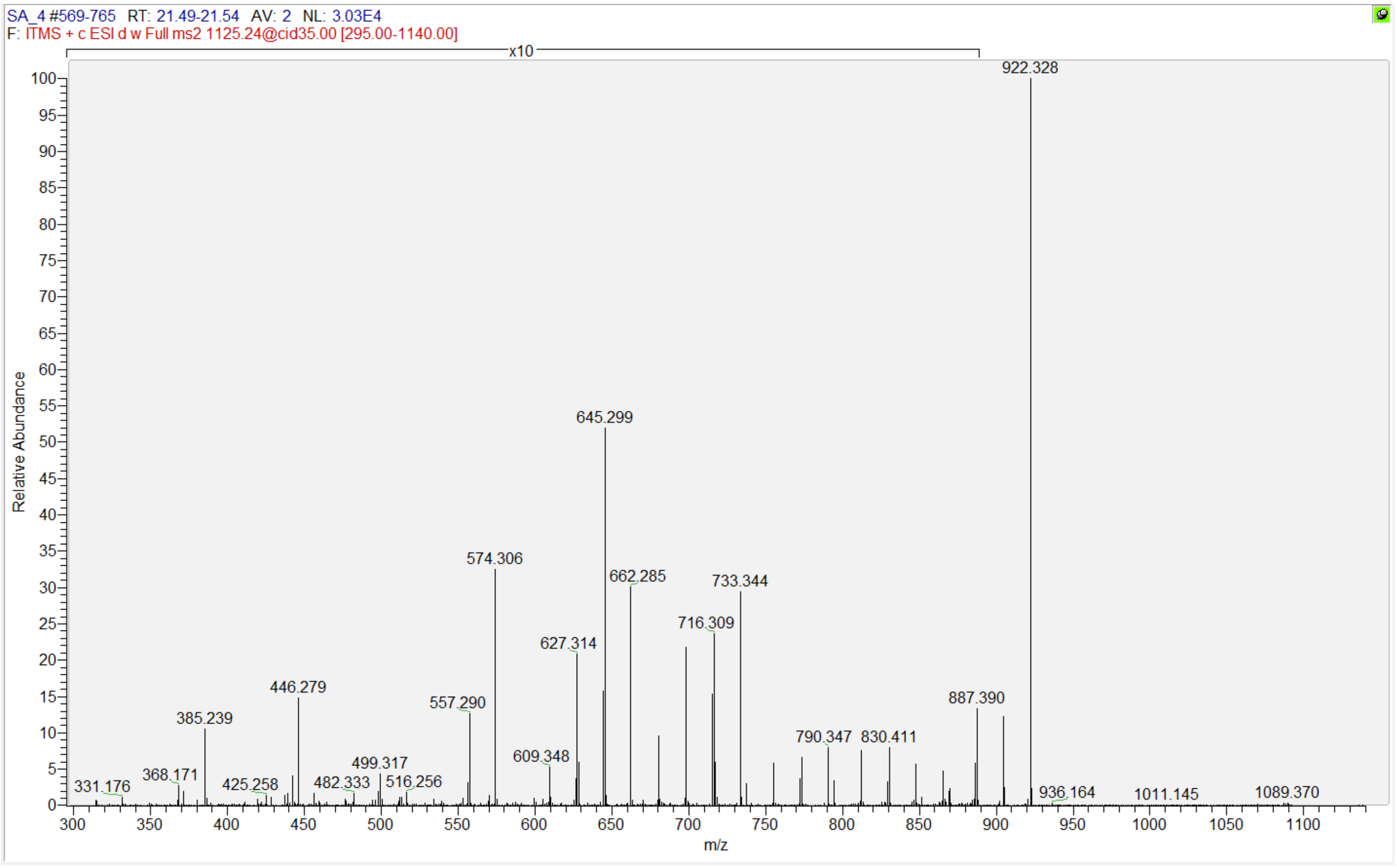

A LC-MS Peak 5

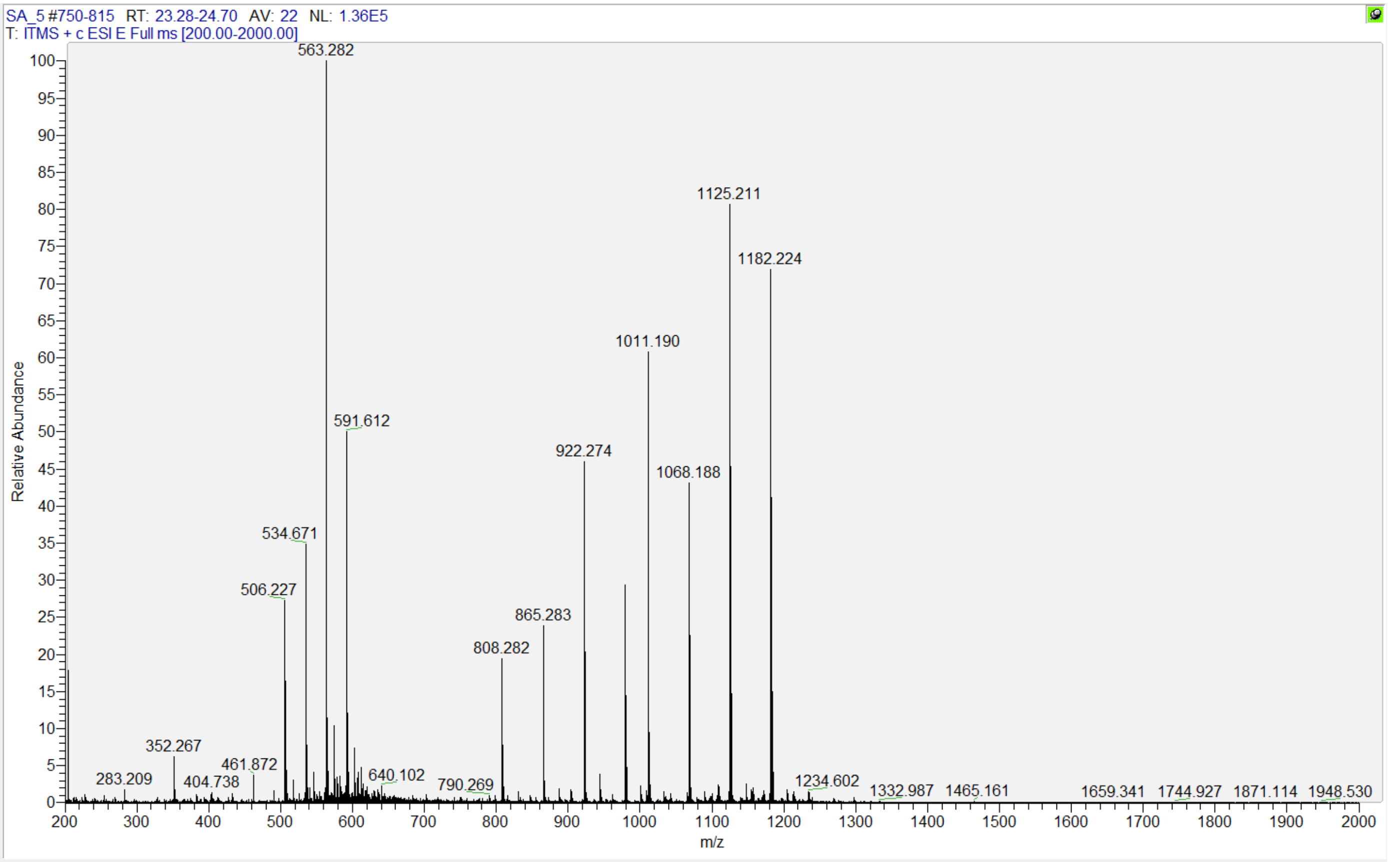

B LC-MS/MS Peak 5

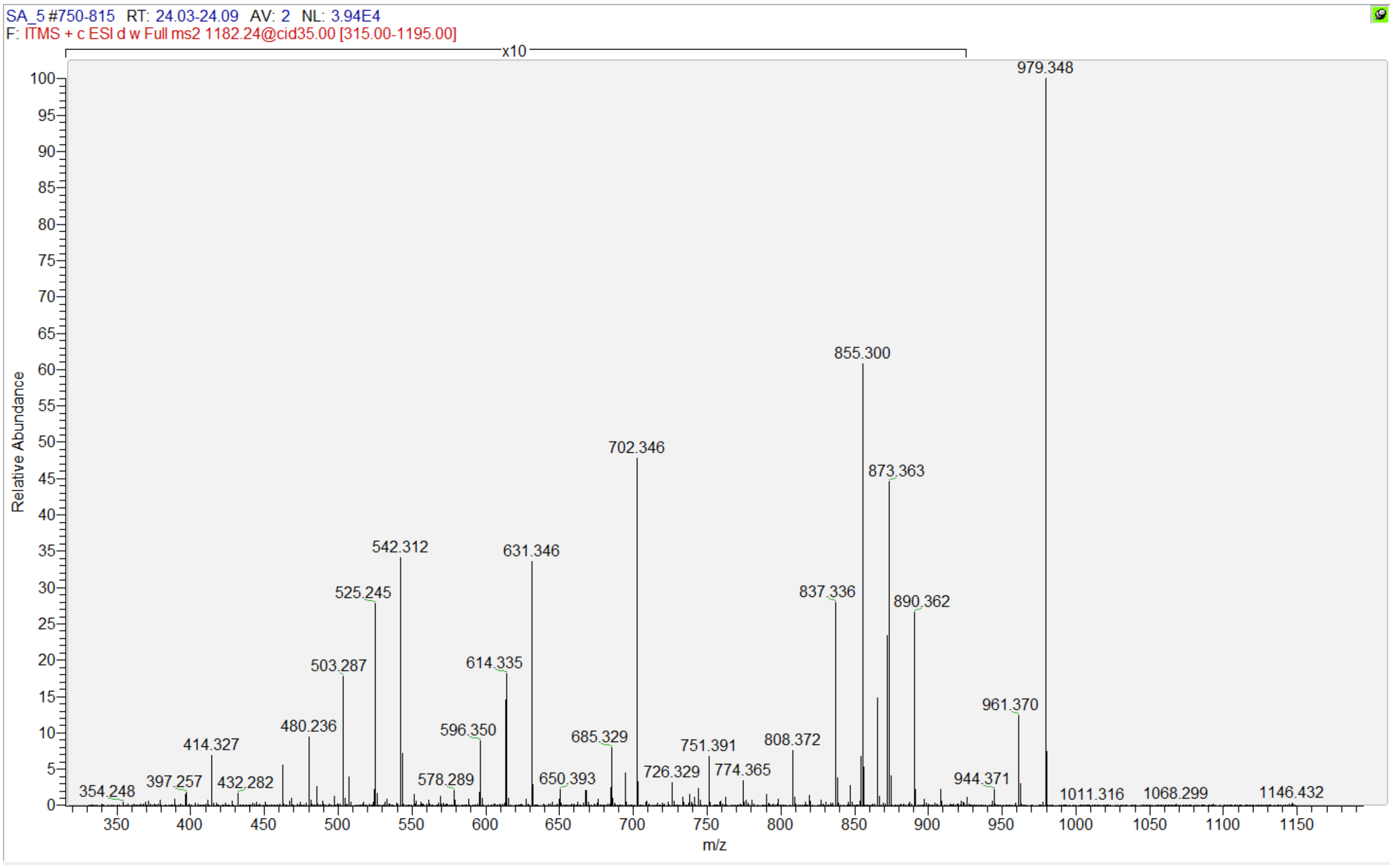

A LC-MS Peak 6

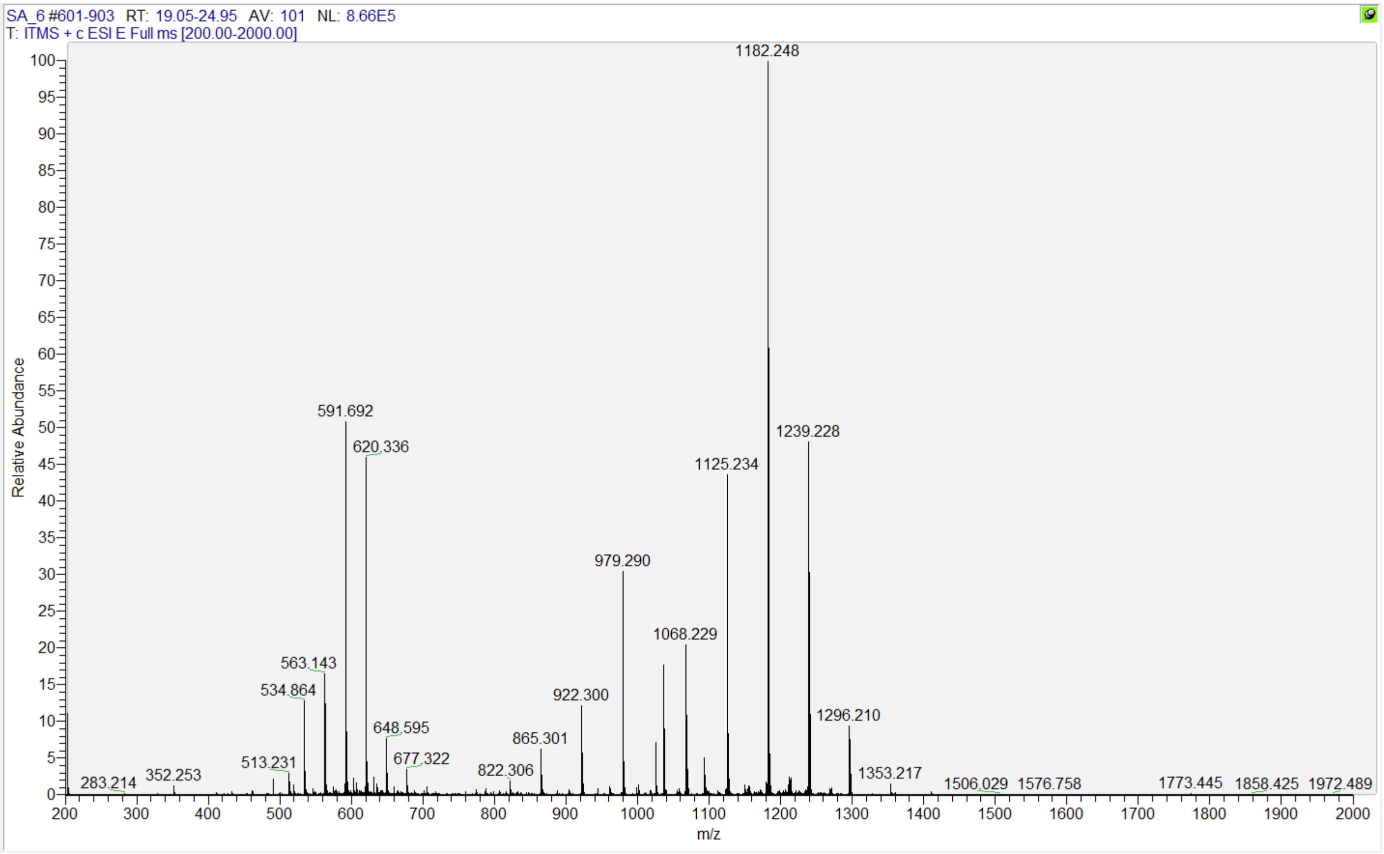

B LC-MS/MS Peak 6

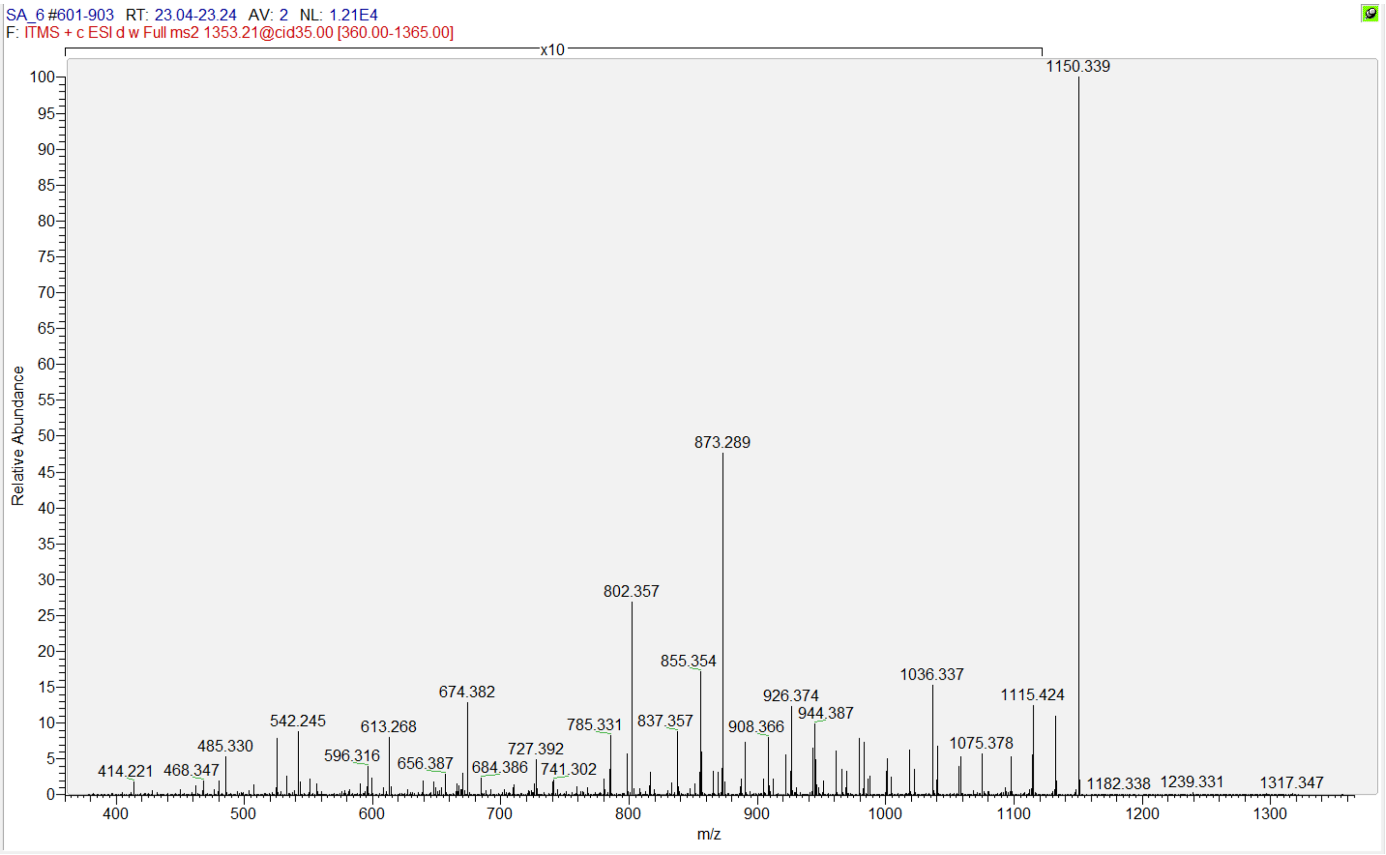

A LC-MS Peak 7

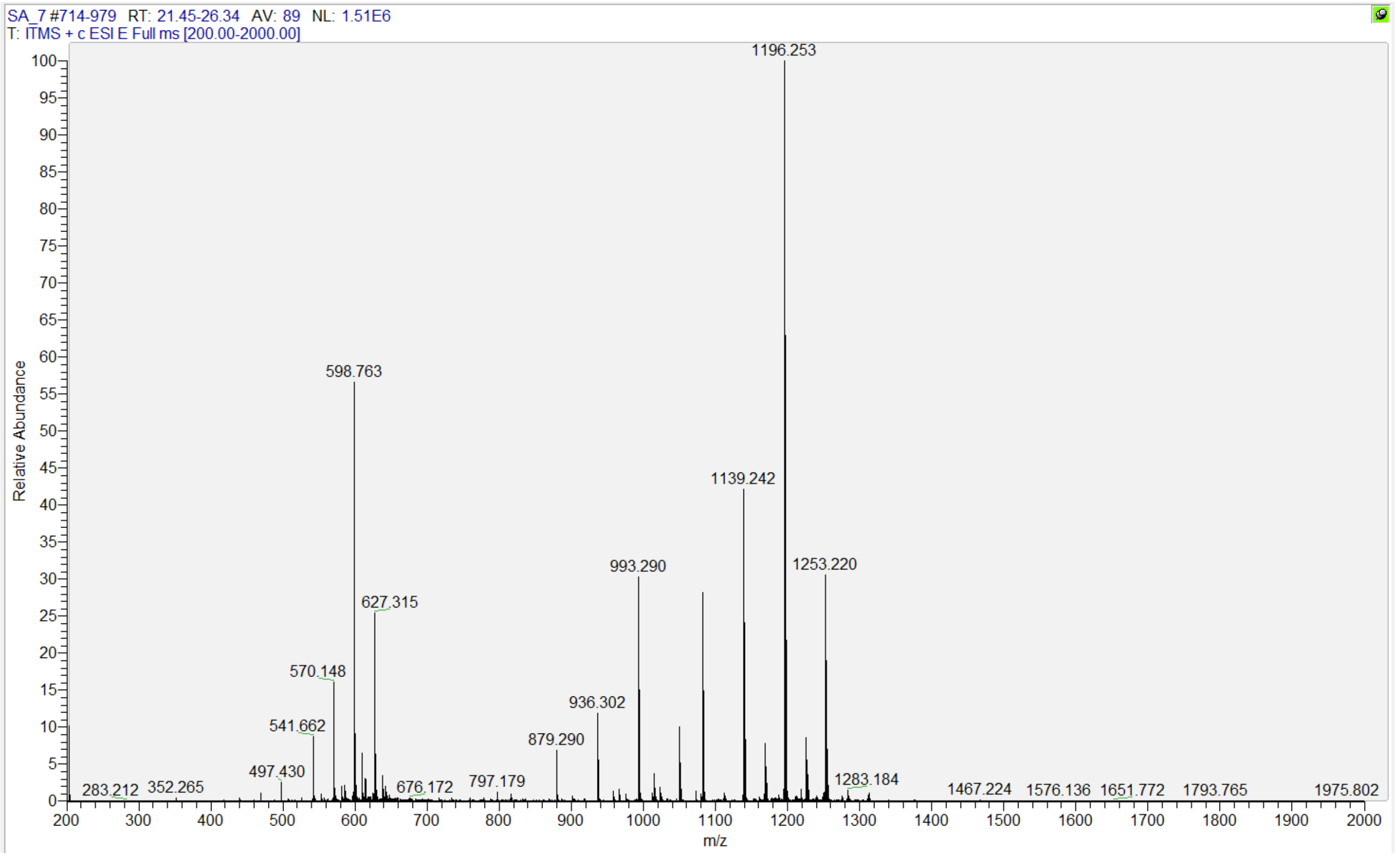

B LC-MS/MS Peak 7

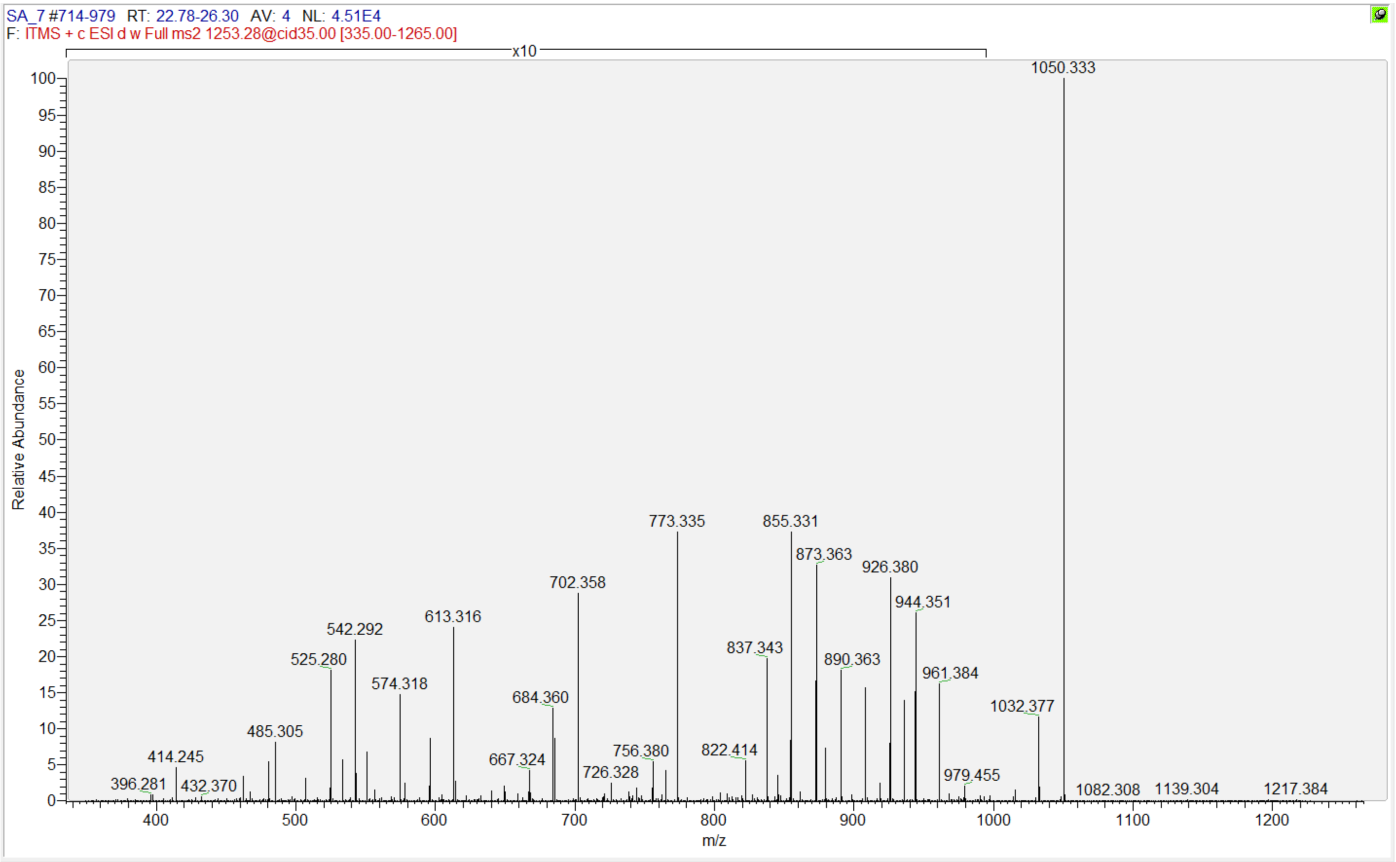

A LC-MS Peak 8

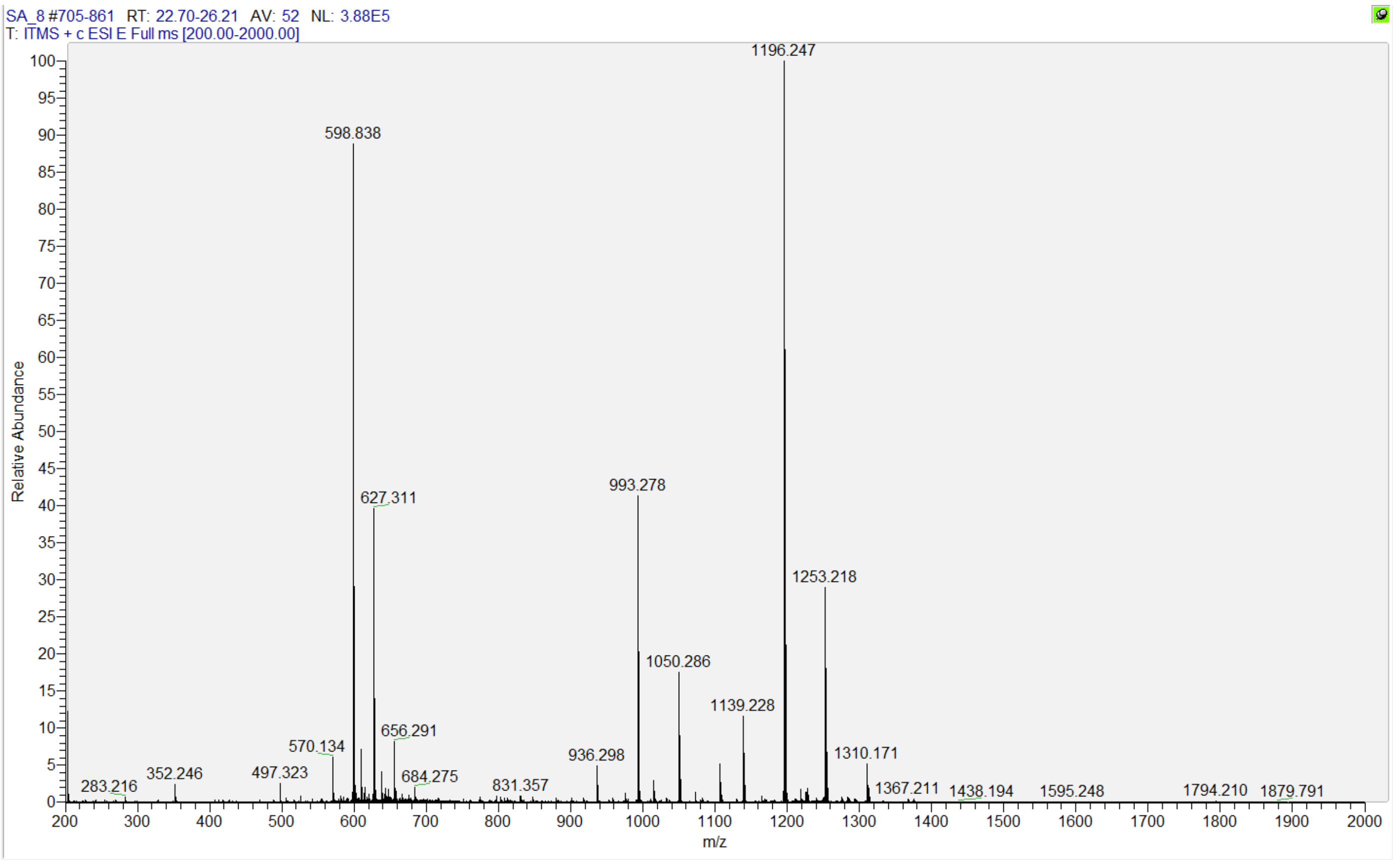

B LC-MS/MS Peak 8

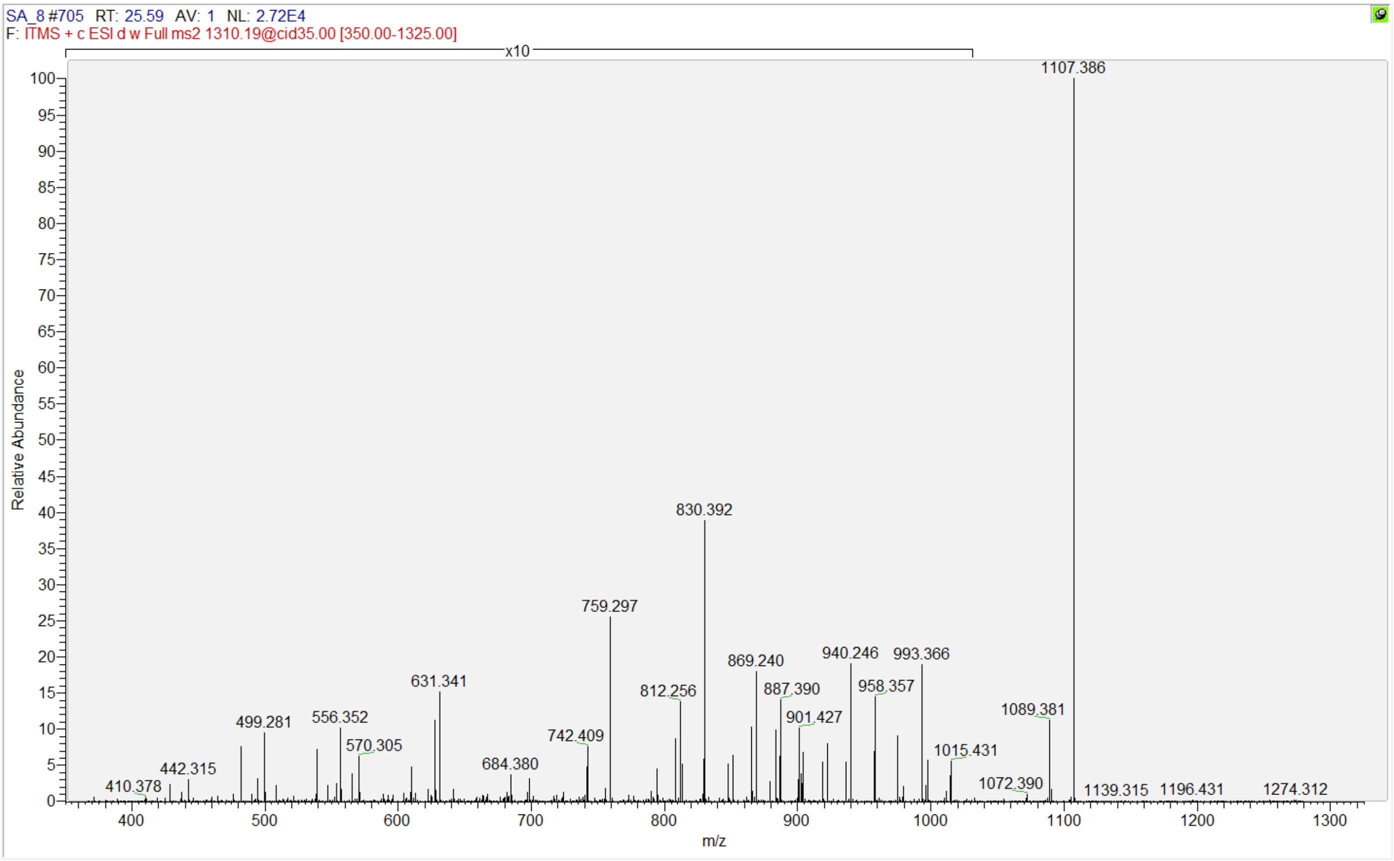

A LC-MS Peak 9

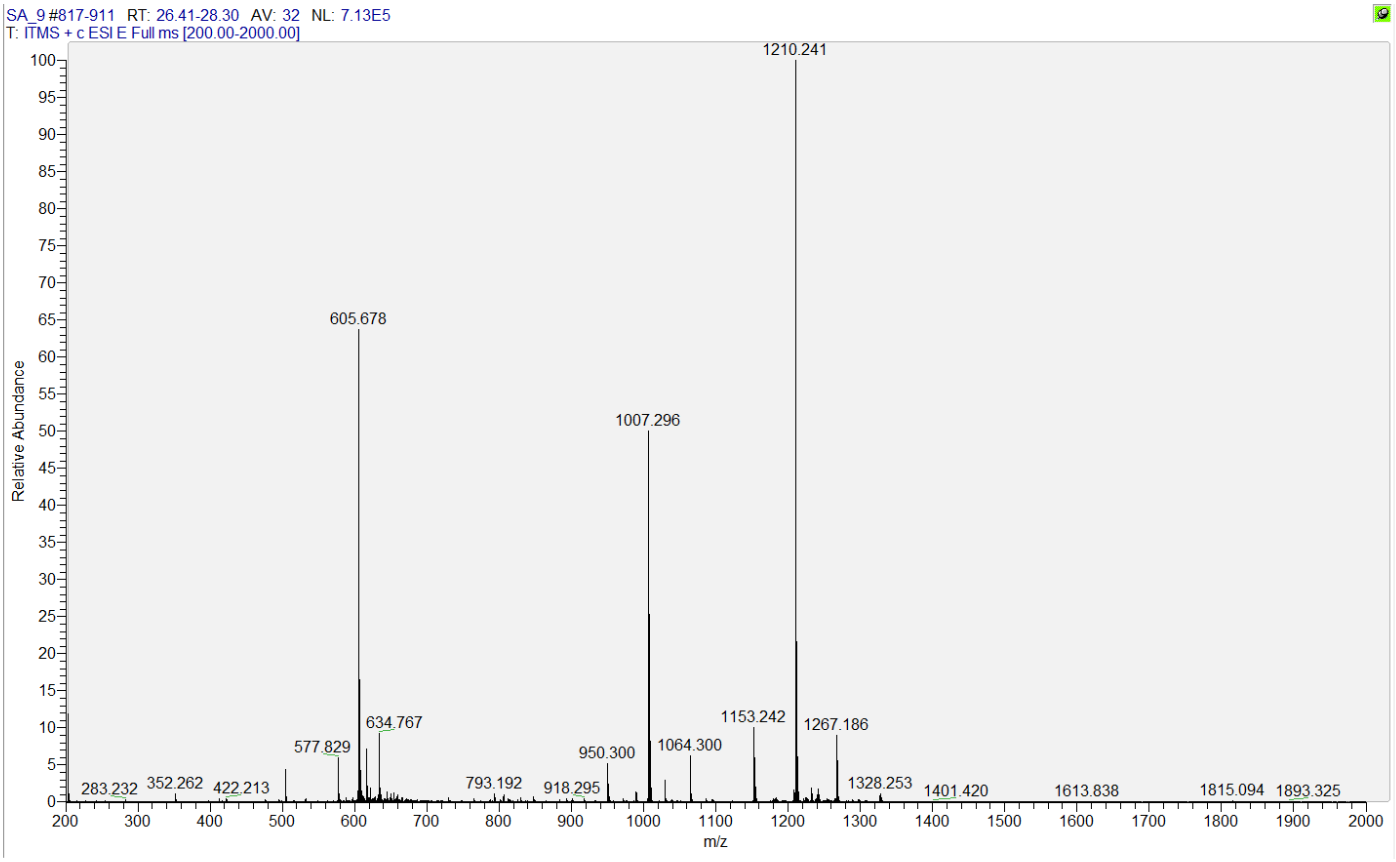

B LC-MS/MS Peak 9

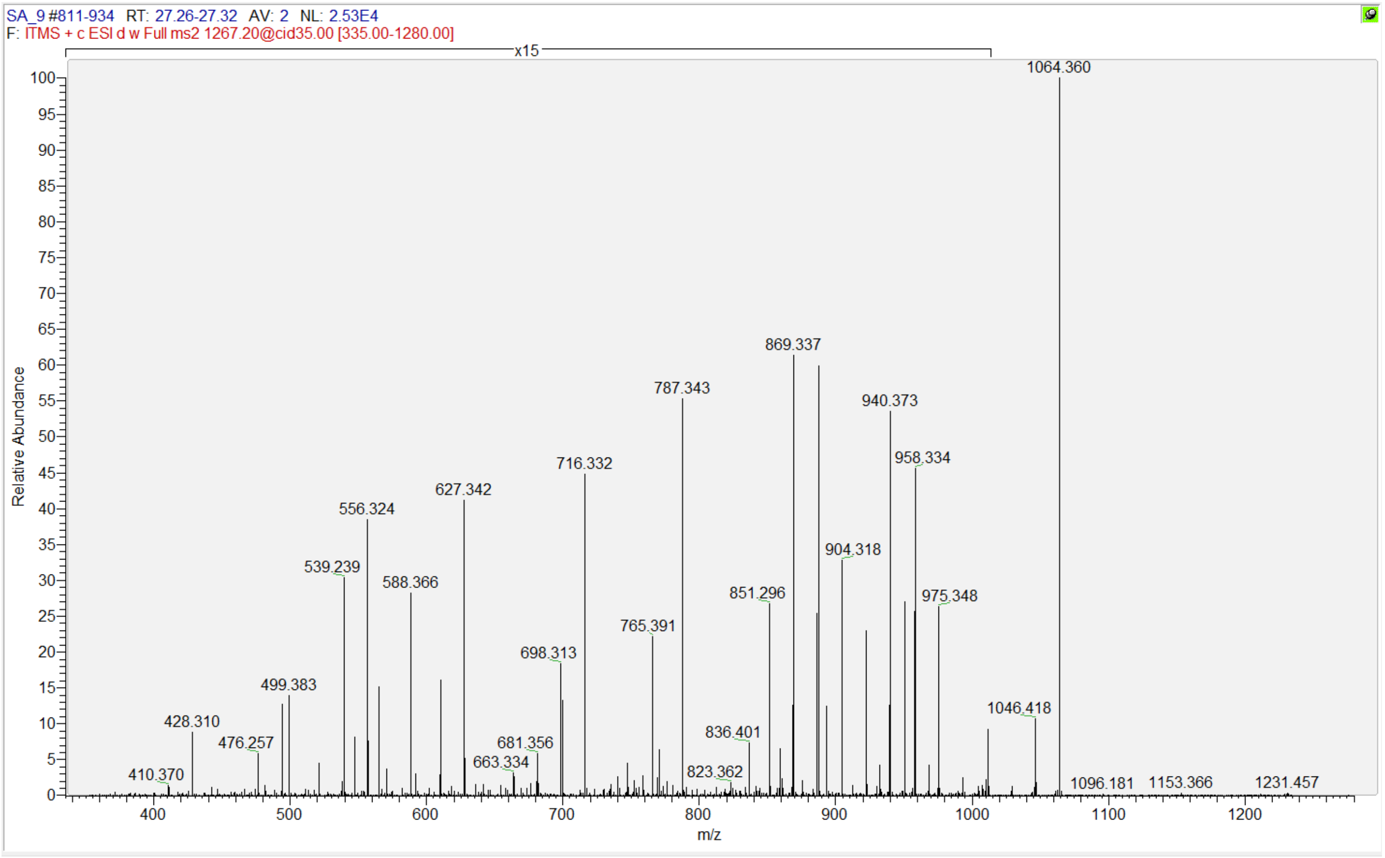

A LC-MS Peak 10

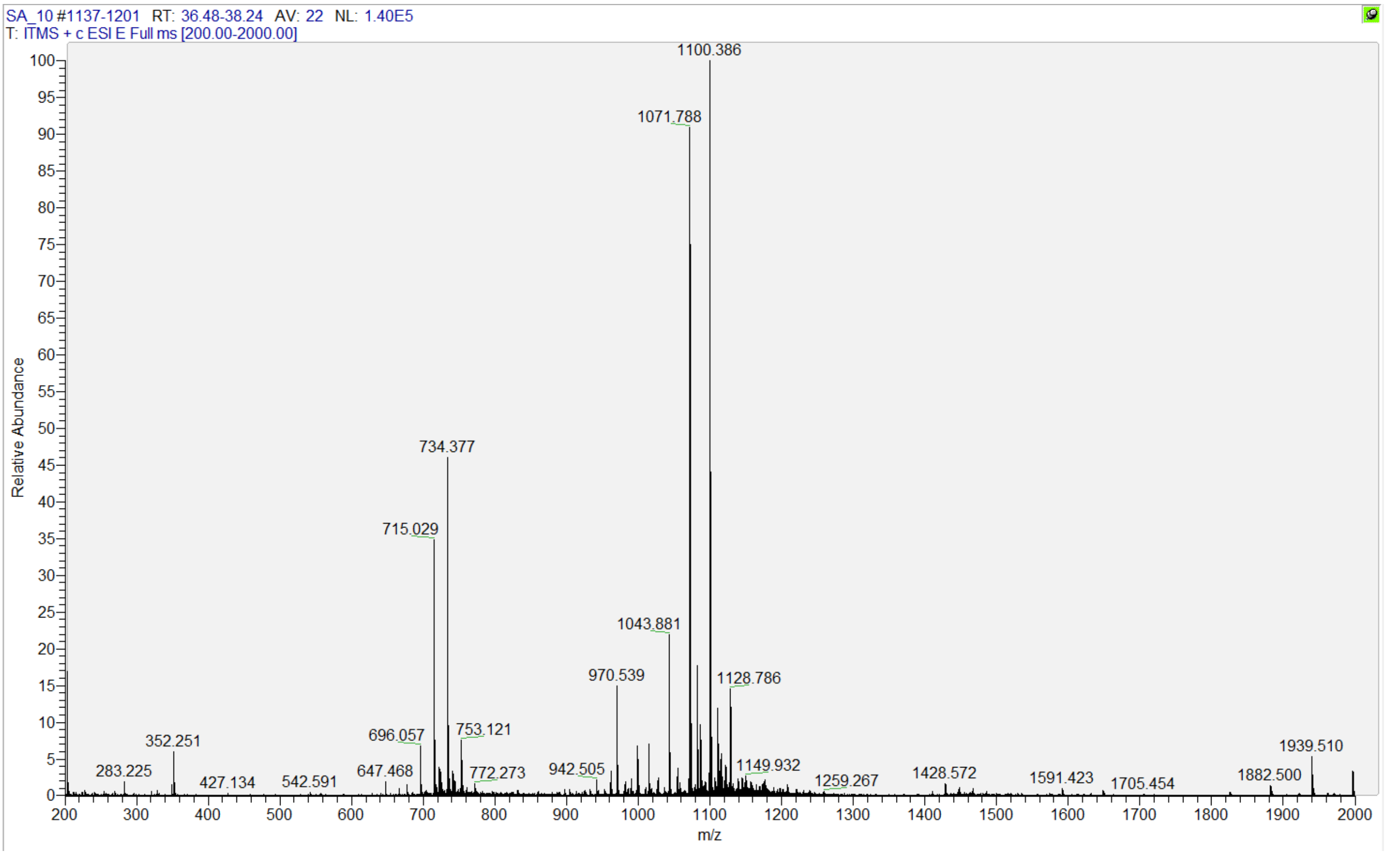

B LC-MS/MS Peak 10

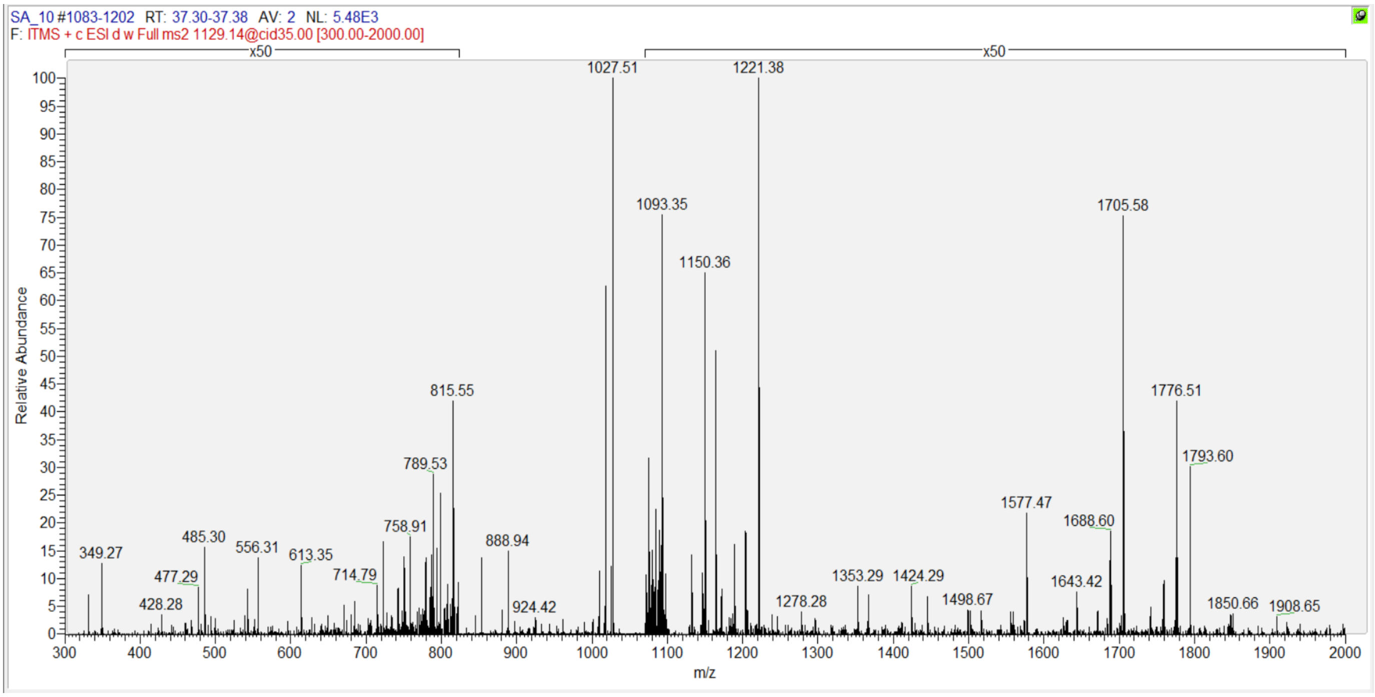

A LC-MS Peak 11

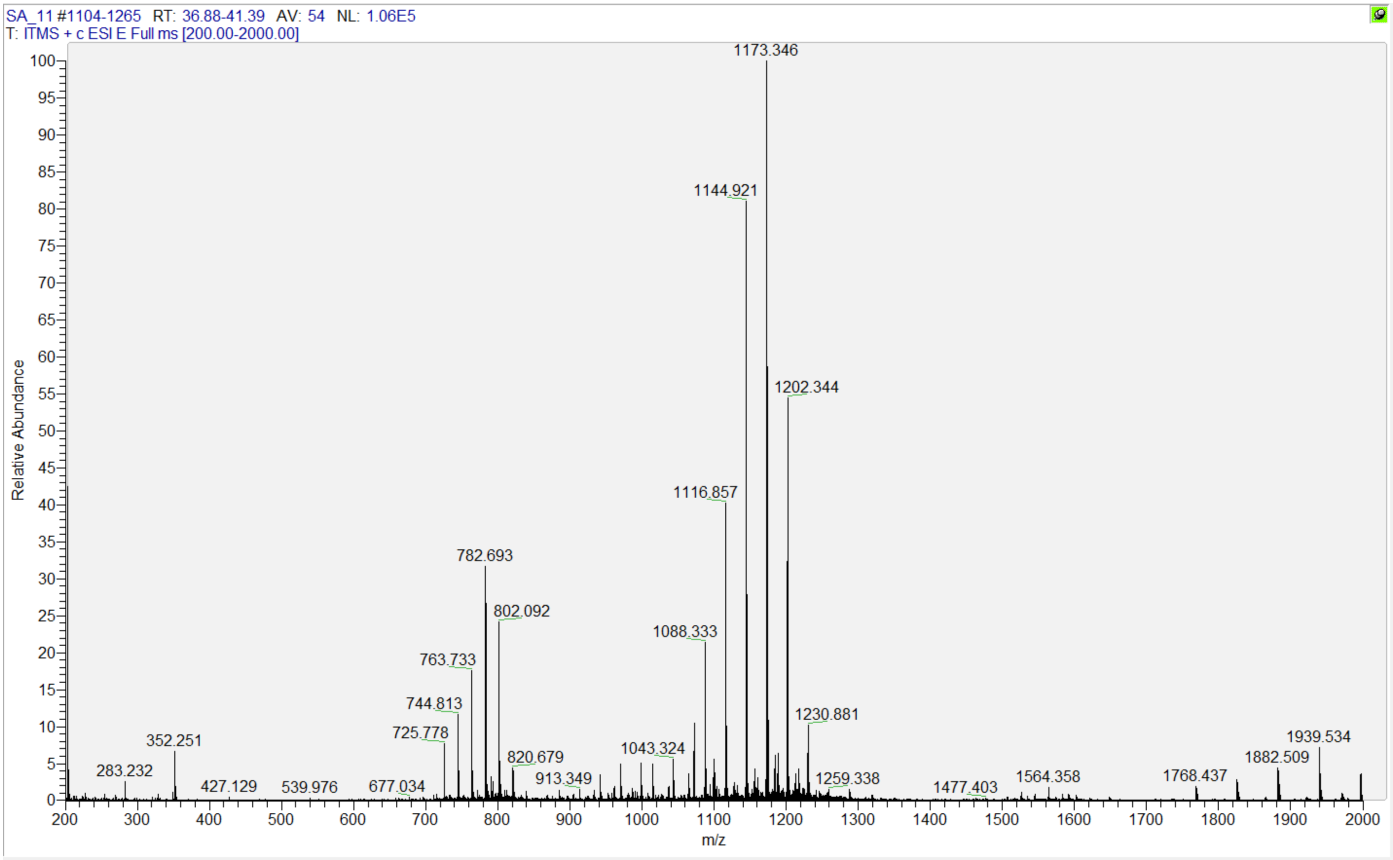

B LC-MS/MS Peak 11

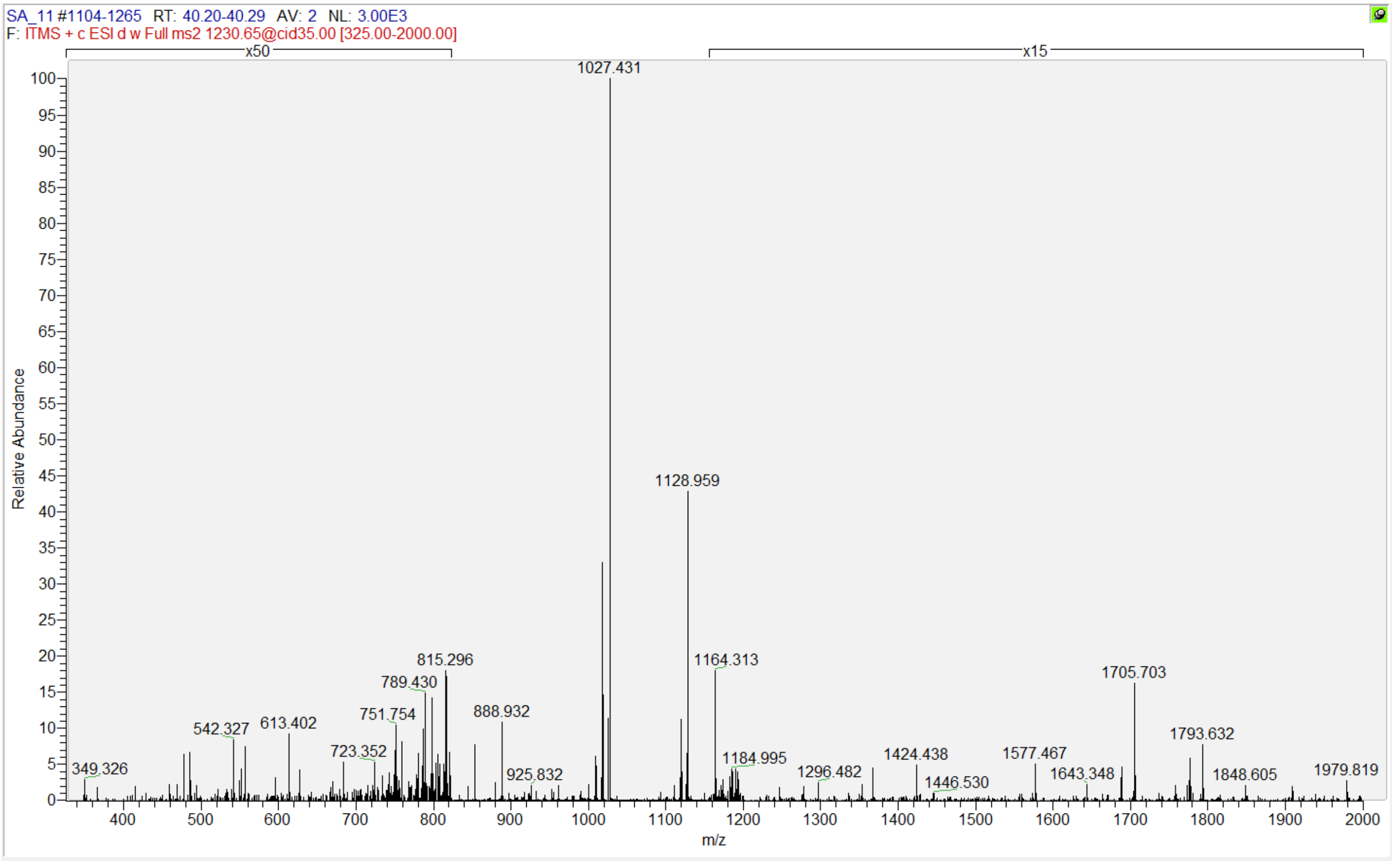

A LC-MS Peak 12

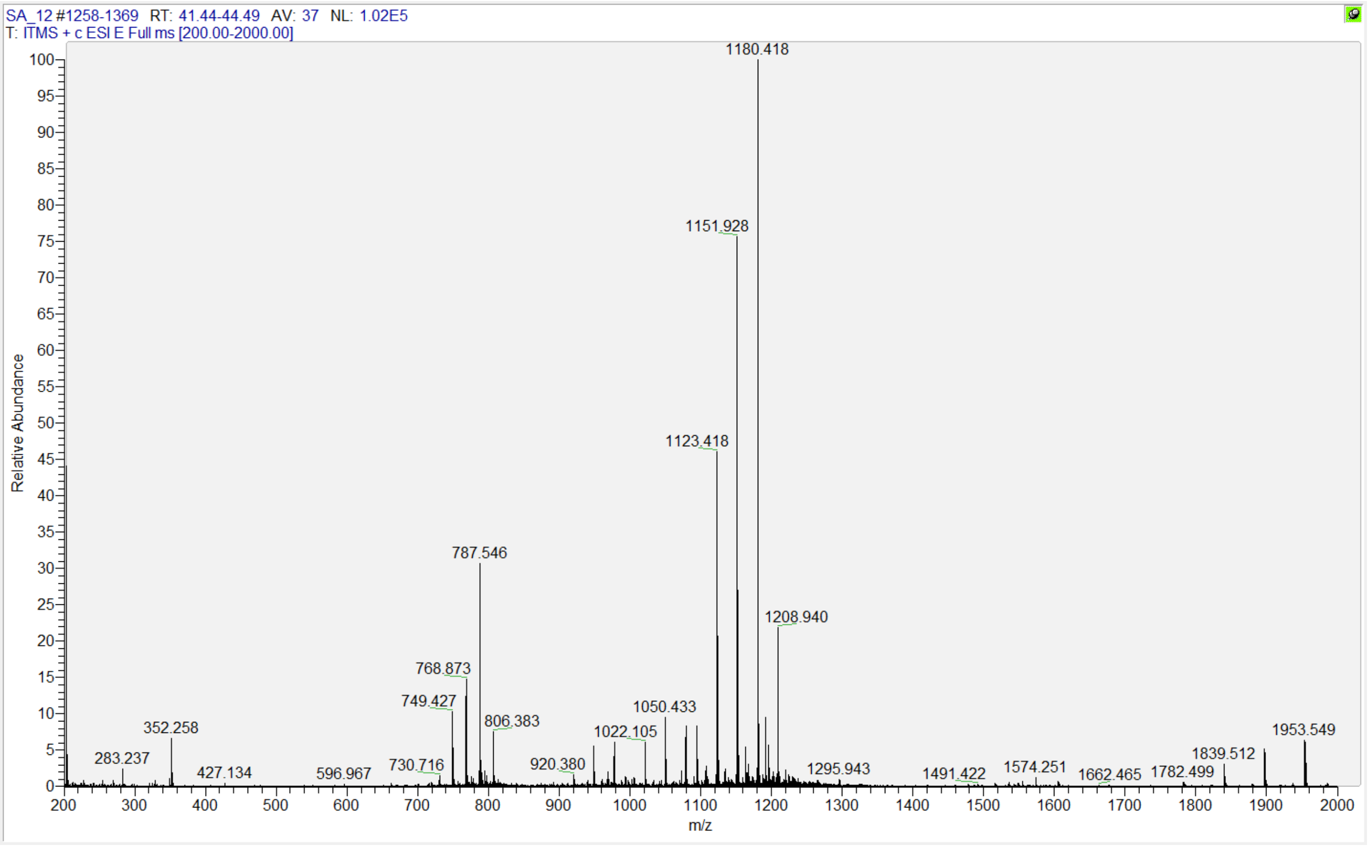

B LC-MS/MS Peak 12

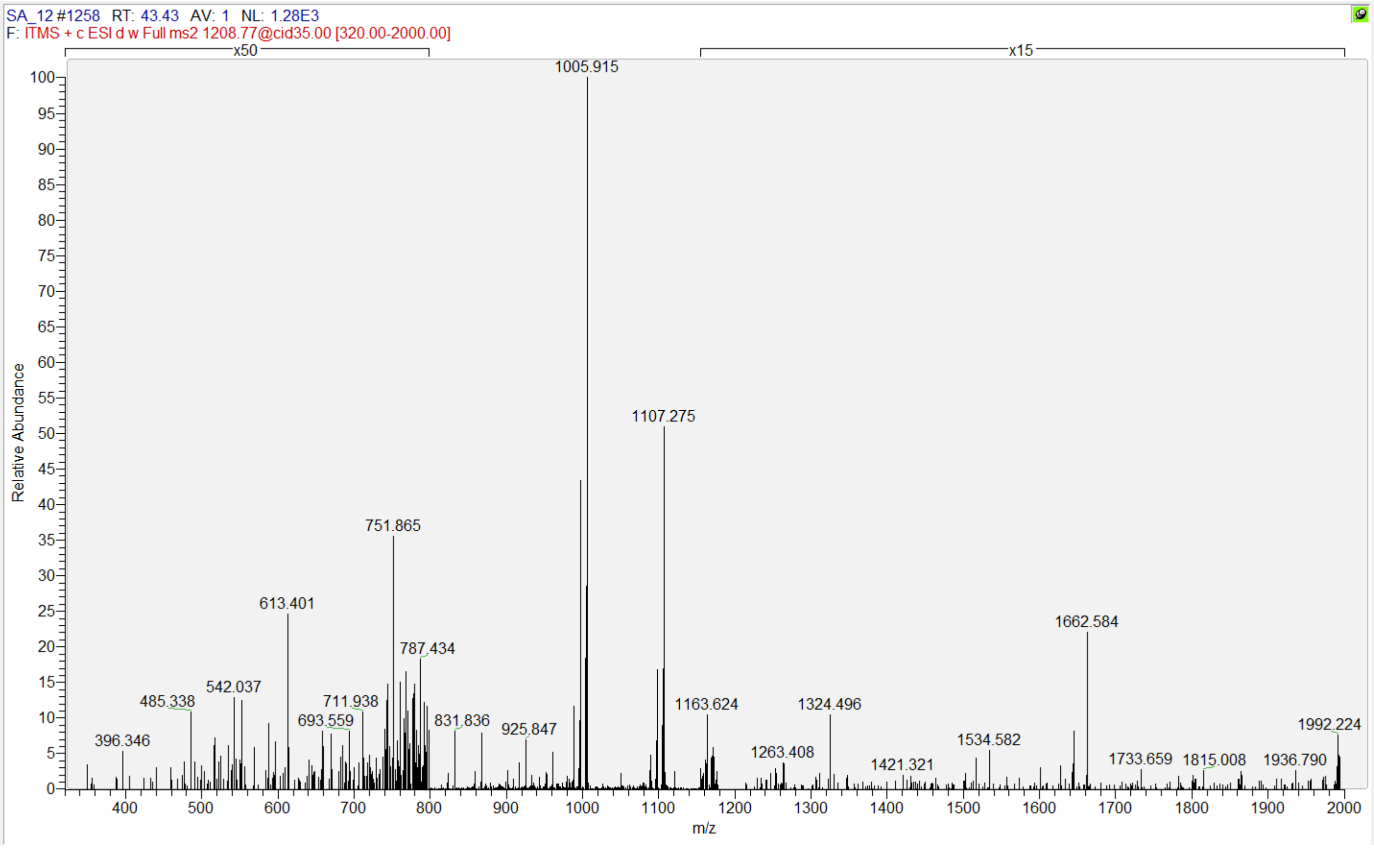

A LC-MS Peak 13

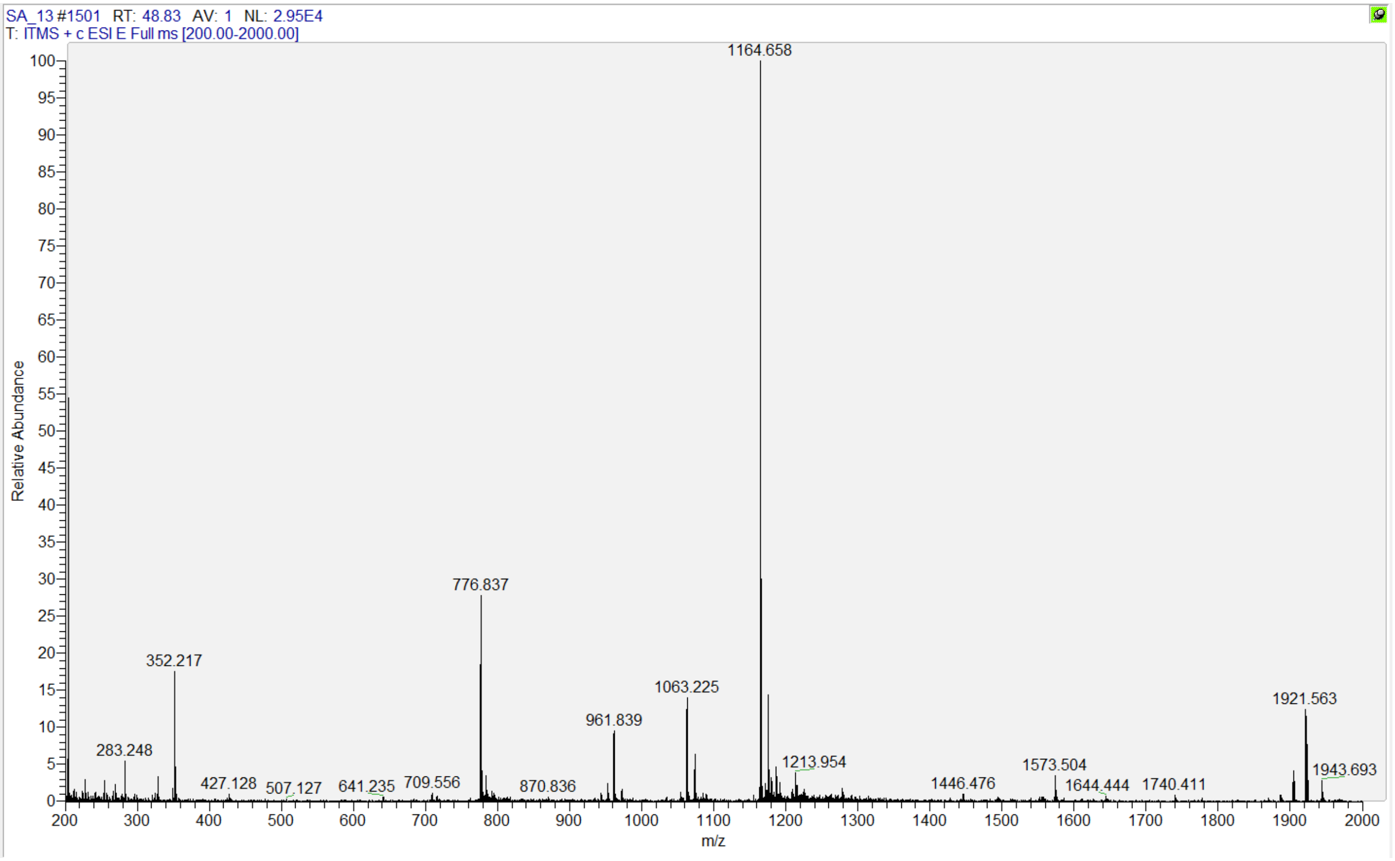

B LC-MS/MS Peak 13

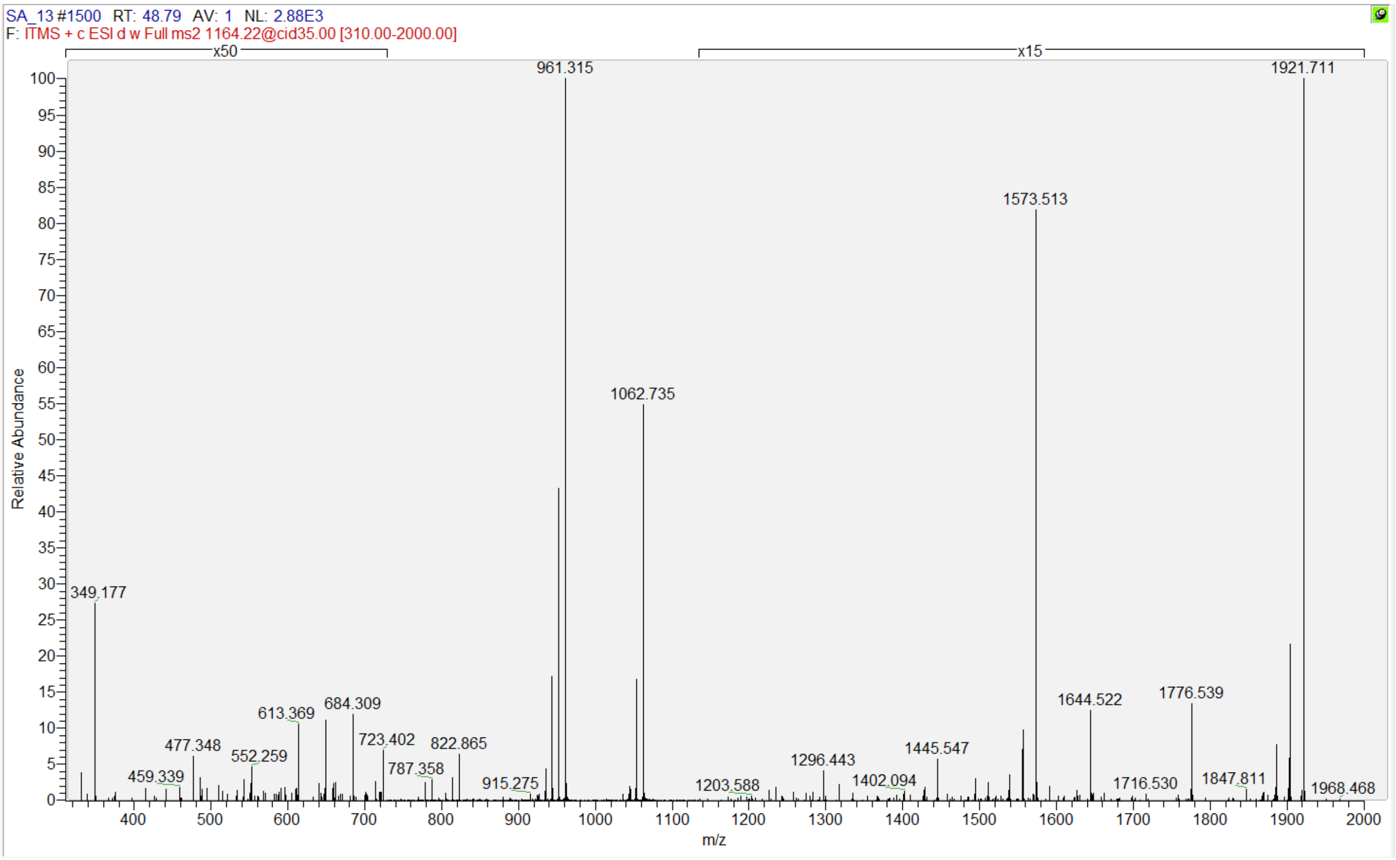

Figure S4: Identification of JE2Δ*mpsABC* PG fragments by LC-MS and LC-MS/MS. The collected peaks from the lysostaphin-cellosyl double-digested Δ*mpsABC* PG profile (Fig. 4a) were analyzed by LC-MS (A) and LC-MS/MS (B). The determined masses of fragmented PG are summarized in Table S3, while the corresponding identified fragments are illustrated in Fig. 4b.
