# Supplementary material for: Staphylococcus aureus Stress Response to Bicarbonate Depletion
Source: Int J Mol Sci. 2024 Aug 26;25(17):9251. doi: 10.3390/ijms25179251 (PMC11394868; doi:10.3390/ijms25179251)
Supplement: Supplementary file 1 [file ijms-25-09251-s001.zip › Table S1.docx]

**Table S1: The release of genomic DNA (gDNA) upon cell lysis from JE2 parent strain and Δ*mpsABC.***

|  |  | gDNA concentration (ng/µL ± SD) | |
| --- | --- | --- | --- |
|  |  | Strain | |
| **Lysostaphin** (µg/mL) | **Condition** | ***S. aureus* JE2 parent** | ***S. aureus* JE2∆*mpsABC*** |
| 50 | ambient air | 84.4 ± 13.1 | 11.1 ± 0.7 |
|  | 5% CO_2_ | 76.8 ± 13.0 | 45.5 ± 5.3 |
| 500 | ambient air | 75.7 ± 16.3 | 15.5 ± 0.4 |
|  | 5% CO_2_ | 62.4 ± 2.4 | 69.2 ± 12.8 |

Concentration of gDNA upon 30 min treatment with lysostaphin. gDNA was isolated using *Quick*- DNA^TM^ Microprep Kit (ZYMO Research Europ GmbH, Freiburg, GER) and measured using a NanoPhotometer® NP80 (Implen GmbH, München, GER). Each value represents the mean ± standard deviations (SD) from at least three independent biological replicates.
