# Supplementary material for: Staphylococcus aureus Stress Response to Bicarbonate Depletion
Source: Int J Mol Sci. 2024 Aug 26;25(17):9251. doi: 10.3390/ijms25179251 (PMC11394868; doi:10.3390/ijms25179251)
Supplement: Supplementary file 1 [file ijms-25-09251-s001.zip › Table S2.docx]

**Table S2: The percentage distribution of mutanolysin digested muropeptides.**

|  | Percentage of muropeptides (mean ± SD %) | | | | | | |
| --- | --- | --- | --- | --- | --- | --- | --- |
| Growth condition | Strain | Monomer | Dimer | Trimer | Tetramer | Pentamer | Hexamer |
| Ambient air | *S. aureus* JE2 parent | 15.1 ± 1.7 | 21.5 ± 4.1 | 22.9 ± 1.4 | 12.6 ± 0.4 | 13.4 ± 0.2 | 14.4 ± 0.4 |
|  | *S. aureus* JE2Δ*mpsABC* | 16.5 ± 1.3 | 15.2 ± 8.2 | 24.8 ± 3.5 | 13.6 ± 1.6 | 14.4 ± 1.4 | 15.5 ± 1.6 |
| 5% CO2 | *S. aureus* JE2 parent | 12.9 ± 1.2 | 22.4 ± 5.1 | 20.8 ± 5.6 | 11.8 ± 3.2 | 15.5 ± 2.0 | 16.6 ± 1.9 |
|  | *S. aureus* JE2Δ*mpsABC* | 11.5 ± 3.9 | 20.0 ± 0.6 | 24.8 ± 0.7 | 13.8 ± 0.7 | 14.5 ± 1.1 | 15.5 ± 0.9 |

Bacteria were grown in ambient air or 5% CO2 conditions until A578 0.6 - 0.8. The peptidoglycan was purified and digested with 200 Units of mutanolysin (Sigma-Aldrich Co., St.Louis, MO (USA). Digested muropeptides were analyzed by RP-HPLC. Muropeptide peaks of the chromatogram were set in percent relation with the total area. The percentage of distribution are three individual RP-HPLC measurements of independent biological replicates.
