# Supplementary material for: Staphylococcus aureus Stress Response to Bicarbonate Depletion
Source: Int J Mol Sci. 2024 Aug 26;25(17):9251. doi: 10.3390/ijms25179251 (PMC11394868; doi:10.3390/ijms25179251)
Supplement: Supplementary file 1 [file ijms-25-09251-s001.zip › Table S3.docx]

**Table S3: Calculated masses by LC-MS/MS of JE2Δ*mpsABC*** **collected muropeptide fragments.**

| **Peak No.** | **Observed M/Z** | **Neutral Mass (calc.)** | **Possible ID (from MS/MS)** |
| --- | --- | --- | --- |
| **1** | 979.24 (1+) | 978.24 | Tetra (Ala-Gln-Lys-Ala)-Gly_5_ (less GlcNAc) |
| **2** | 1240.22 (1+) | 1239.22 | Tetra (Ala-Gln-Lys-Ala)-Gly_6_ |
| **3** | 1036.30 (1+) | 1035.30 | Tetra (Ala-Gln-Lys-Ala)-Gly_6_ (less GlcNAc) |
| **4** | 1125.21 (1+) | 1124.21 | Tetra (Ala-Gln-Lys-Ala)-Gly_4_ |
| **5** | 1182. 22 (1+) | 1181.22 | Tetra (Ala-Gln-Lys-Ala)-Gly_5_ |
| **6** | 1353.22 (1+) | 1352.22 | Tetra (Ala-Gln-Lys-Ala)-Gly_8_ |
| **7** | 1253.22 (1+) | 1252.22 | Tetra(Ala-Gln-Lys-Ala)-Gly_2_AlaGly_2_ or Penta-Gly_5_ |
| **8** | 1310.17 (1+) | 1309.17 | Penta Gly_6_ |
| **9** | 1267.19 (1+) | 1266.19 | Penta Gly_2_AlaGly_2_ |
| **10** | 1128.79 (2+) | 2255.58 | Tetra (Ala-Gln-Lys-Ala)-Gly_5_-Tetra (Gly_7_) (less GlcNAc) |
| **11** | 1230.88 (2+) | 2459.76 | Tetra (Ala-Gln-Lys-Ala)-Gly_5_-Tetra (Gly_7_) |
| **12** | 1208.94 (2+) | 2415.88 | Tetra (Ala-Gln-Lys-Ala)-Gly_2_AlaGly_2_-Tetra (Gly_7_) |

The purified PG of JE2Δ*mpsABC* was digested with lysostaphin and cellosyl. The peaks collected from the digested PG (Fig. 4A) were determined by LC-MS/MS (Fig. S4). The predicted structures of the muropeptides are shown in Fig. 4B.
