# Supplementary material for: Staphylococcus aureus Stress Response to Bicarbonate Depletion
Source: Int J Mol Sci. 2024 Aug 26;25(17):9251. doi: 10.3390/ijms25179251 (PMC11394868; doi:10.3390/ijms25179251)
Supplement: Supplementary file 1 [file ijms-25-09251-s001.zip › Table S4.docx]

**Table S4: Oligonucleotides used in qRT-PCR.**

| **Reference number** | **Primer** | **Sequence (5’→ 3’)** |
| --- | --- | --- |
| B7H15_00025 | *gyrB* - fwd | CGCAGGCGATTTTACCATTA |
|  | *gyrB* - rev | GCTTTCGCTAGATCAAAGTCG |
| B7H15_01540 | *esxA* - fwd | GTCAAGCTTTCAGCCGTTTC |
|  | *esxA* - rev | CTTGTTCTTGAACGGCATCAG |
| B7H15_00750 | *sasD* - fwd | GGAATGACGCAAGCACAATATAC |
|  | *sasD* - rev | TTGCGTCGCATCATACAATTTC |
| B7H15_06050 | *hla* - fwd | AGTCCAGTGCAATTGGTAGTC |
|  | *hla* - rev | CACCAGACTTCGCTACAGTTATT |
| B7H15_11645 | *sceD* - fwd | GCGGCATCTTGAACACTTTC |
|  | *sceD* - rev | GTGCAGCTGGTAAGTATCAATTC |
| B7H15_01500 | *lytM* - fwd | GTCCAGACGCGAGCTATTATT |
|  | *lytM* - rev | CCACTAGCTGTCGCTTTACTT |
